# Expression of a functional oxygen-labile nitrogenase component in the mitochondrial matrix of aerobically grown yeast

**DOI:** 10.1038/ncomms11426

**Published:** 2016-04-29

**Authors:** Gema López-Torrejón, Emilio Jiménez-Vicente, José María Buesa, Jose A. Hernandez, Hemant K. Verma, Luis M. Rubio

**Affiliations:** 1 Centro de Biotecnología y Genómica de Plantas, Universidad Politécnica de Madrid, Pozuelo de Alarcón, Madrid 28223, Spain; 2 Department of Biochemistry, Midwestern University, Glendale, Arizona 85308, USA

## Abstract

The extreme sensitivity of nitrogenase towards oxygen stands as a major barrier to engineer biological nitrogen fixation into cereal crops by direct *nif* gene transfer. Here, we use yeast as a model of eukaryotic cell and show that aerobically grown cells express active nitrogenase Fe protein when the NifH polypeptide is targeted to the mitochondrial matrix together with the NifM maturase. Co-expression of NifH and NifM with Nif-specific Fe–S cluster biosynthetic proteins NifU and NifS is not required for Fe protein activity, demonstrating NifH ability to incorporate endogenous mitochondrial Fe–S clusters. In contrast, expression of active Fe protein in the cytosol requires both anoxic growth conditions and co-expression of NifH and NifM with NifU and NifS. Our results show the convenience of using mitochondria to host nitrogenase components, thus providing instrumental technology for the grand challenge of engineering N_2_-fixing cereals.

## Abstract (web summary)

The sensitivity of nitrogenase to oxygen is a major barrier to engineer biological nitrogen fixation into cereal crops by direct *nif* gene transfer. Here the authors use yeast to show that targeting nitrogenase Fe protein to the mitochondrial matrix overcomes the O_2_ sensitivity impediment.

## Graphical abstract

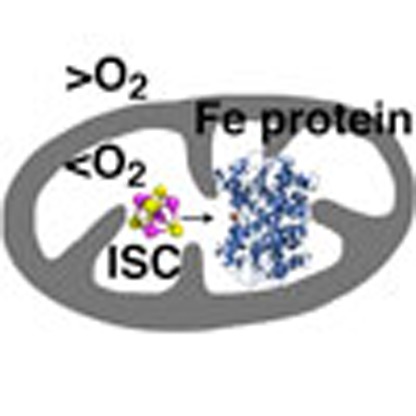

Productivity of major crops is often limited by availability of fixed nitrogen sources such as NH_3_ and nitrate^1^. For the last 100 years, the addition of chemically synthesized nitrogen fertilizers has sustained crop productivity, but at significant environmental and economic costs^2^,^3^. An alternative sustainable solution to this problem is the incorporation of biological nitrogen fixation into cereal crops, for which transfer of bacterial *nif* genes into the plant is one possible strategy^4^,^5^,^6^.

Mo-nitrogenase is composed of two metalloproteins: the *nifDK*-encoded dinitrogenase component (also termed MoFe protein) that catalyses the reduction of N_2_ to NH_3_, and the *nifH*-encoded dinitrogenase reductase (also termed Fe protein) that is the obligate electron donor to the MoFe protein^7^. Additional gene products (minimally NifB, NifE and NifN) are required for the assembly of nitrogenase metal cofactors^8^,^9^. Both nitrogenase components, as well as most proteins required for the assembly of their metal clusters, are very sensitive to O_2_ (refs 10, 11, 12). The initial hypothesis of this work was that the mitochondrial matrix could provide a low O_2_ environment appropriate for the assembly and the activity of nitrogenase components similarly to the *Azotobacter vinelandii* respiratory protection^13^. The Fe protein was chosen to obtain proof of concept because it is more sensitive to O_2_ than the MoFe protein^14^ and because its maturation requirements are simpler^5^. In model diazotrophs *Klebsiella pneumoniae* and *A. vinelandii*, NifH polypeptides require NifM, NifS and NifU to render active Fe protein^15^,^16^. NifM is a putative peptidyl-prolyl isomerase^17^, whose exact role on NifH maturation has not been established. NifS is a cysteine desulfurase that provides S for formation of Fe–S clusters on the scaffolding protein NifU^18^,^19^, which in turn transfers [4Fe–4S] clusters to apo-NifH generating mature Fe protein^20^. Here we show that targeting nitrogenase Fe protein to the mitochondrial matrix overcomes the O_2_ sensitivity problem, thus breaking through a major barrier to produce active nitrogenase within a eukaryotic cell.

## Results and Discussion

### Targeting of Nif proteins to yeast mitochondria

**Figure 1:**
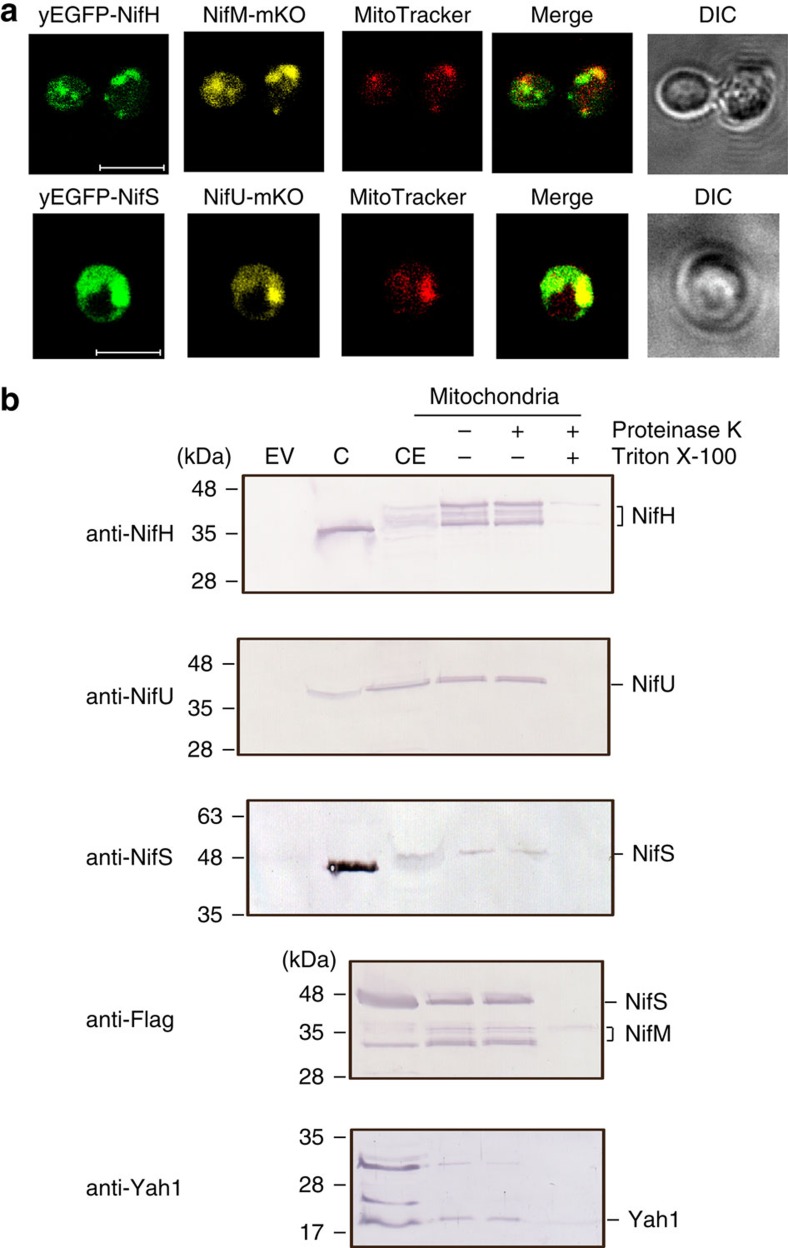
Mitochondrial targeting of Nif proteins. (**a**) Confocal microscopy images of *S. cerevisiae* strains carrying synthetic *sod2*-*yEGFP*-*nifH* and *sod2*-*nifM*-*mKO* (strain GF5) or *sod2*-*yEGFP*-*nifS* and *sod2*-*mKO-nifU* genes (strain GF7), which expression was induced by galactose in the growth medium. The mitochondria of galactose-induced cells were localized with MitoTracker Deep Red. Scale bar, 5 μm. (**b**) Immunoblot analysis of isolated mitochondria developed with antibodies against NifH, NifU, NifS, Yah1 or FLAG (to detect NifS and NifM at the same time). EV represents cell-free extracts from recombinant yeast carrying pESC-His and pESC-Ura plasmids. CE represents cell-free extracts from recombinant yeast carrying NifH, NifM, NifU and NifS cloned into pESC-His and pESC-Ura plasmids (strain GF8). C represents control lanes with purified Nif proteins from *A. vinelandii*. Isolated mitochondria were treated with proteinase K either in the absence of detergent (for the removal of outer membrane proteins) or in the presence of Triton X-100 (to permeabilize the organelle).

*Saccharomyces cerevisiae* was chosen as model eukaryotic cell for the following reasons: (i) the possibility to set different O_2_ levels during expression of Nif proteins, which allows troubleshooting negative O_2_ effects; (ii) its genetic amenability; and (iii) its mitochondrially located bacterial-like Fe–S cluster assembly machinery, which is the best understood among eukaryotes^21^. *A. vinelandii nifH*, *nifM*, *nifU* and *nifS* genes were codon optimized for *S. cerevisiae*, cloned into expression vectors under the control of either GAL1 or GAL10 promoters, and introduced into *S. cerevisiae* W303-1a by transformation (Supplementary Table 1). GAL promoters were used as a way to induce *nif* gene expression and couple it to obligatory respiratory conditions (with galactose as the sole carbon source). For mitochondrial targeting, either *sod2* (ref. 22) or *su9* (ref. 23) leader sequences were fused to the 5′ end of each *nif* gene (Supplementary Fig. 1). To determine the effectiveness of mitochondrial leader sequences, we carried out intracellular localization experiments by using fluorescence confocal microscopy. For these experiments, NifH and NifS were tagged with yEGFP, while NifM and NifU were tagged with mKO. In galactose-induced cells, Nif proteins co-localized with MitoTracker dye indicating successful targeting to mitochondria (Fig. 1a). SDS–PAGE (polyacrylamide gel electrophoresis) immunoblot analysis of isolated mitochondria confirmed the presence of NifH, NifM, NifU and NifS in the mitochondrial matrix, as proteolytic degradation by Proteinase K treatment was only effective on detergent permeabilized mitochondria (Fig. 1b). In contrast, cytosolic versions of NifH, NifM, NifU and NifS (lacking mitochondrial leader sequences; Supplementary Fig. 1c) were not imported into the mitochondrial matrix.

### Purification of active Fe protein from mitochondria

**Figure 2:**
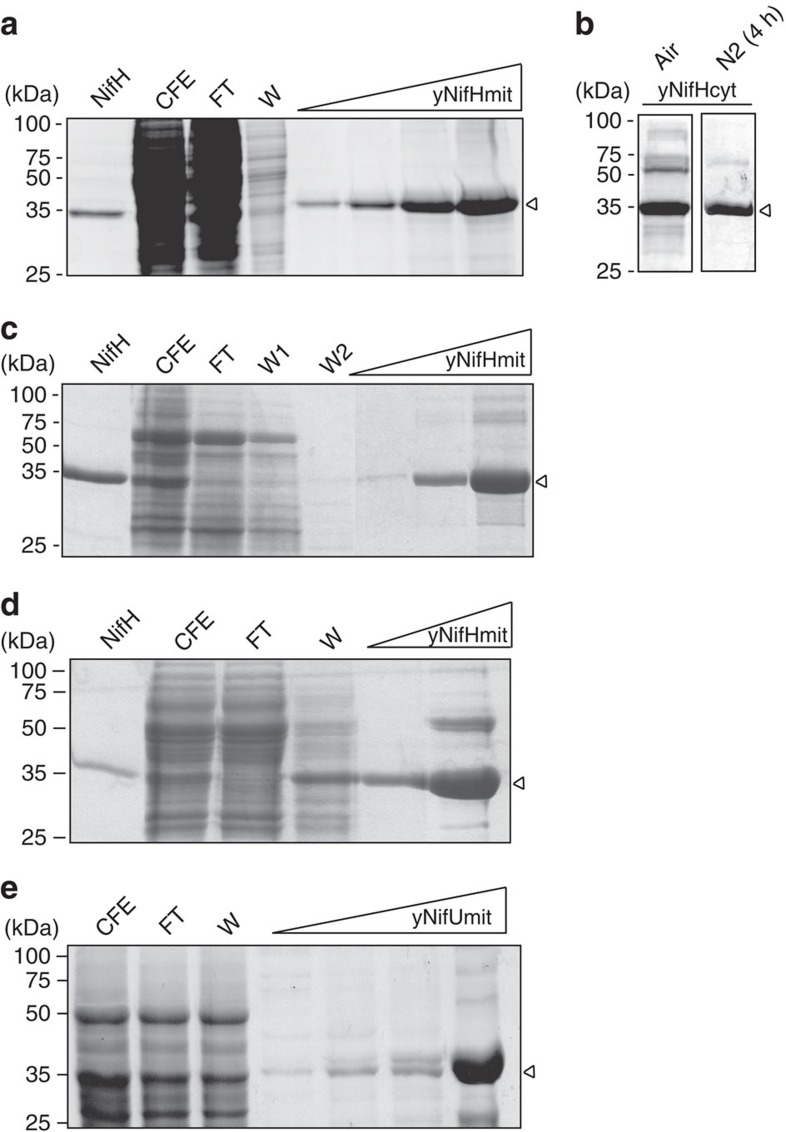
Purification of yNifH and yNifU proteins. Aerated cultures (1 litre air·per minute·per litre of culture) of galactose-induced *S. cerevisiae* cells were used as source of Nif proteins. Co^2+^or Ni^2+^ affinity columns were used for the purification of his-tagged yNifH and yNifU, respectively. The migration of molecular weight markers in SDS–PAGE is indicated at the left side of each panel. Purified *A. vinelandii* NifH was added as control in **a**, **c** and **d**. CFE, cell-free extracts; FT, flow-through fractions; W, protein fractions eluted after washing with washing buffer; yNifHmit, yNifHcyt and yNifUmit indicate protein fractions eluted after applying imidazole to the affinity chromatography columns. Arrows point to purified NifH and NifU proteins. (**a**) yNifHmit purification from GF2 cells co-expressing mitochondria-targeted NifH and NifM. (**b**) yNifHcyt purified from aerated GF9 cells co-expressing cytosol-targeted NifH and NifM that were collected under aerobic conditions (air) or subjected to 4 h of intense N_2_ sparging before cell collection (N_2_). (**c**) yNifHmit purification from GF12 cells, expressing mitochondria-targeted NifH in the absence of NifM. (**d**) yNifHmit purification from GF11 cells co-expressing mitochondria-targeted tagged NifH and NifM along with non-tagged NifU and NifS. (**e**) yNifUmit purification from GF6 cells co-expressing mitochondria-targeted NifU and NifS.

Strongly aerated *S. cerevisiae* cell cultures (1 litre air·per minute·per litre of culture) growing with galactose as carbon source and inducer of *nif* gene expression were used to produce recombinant Nif proteins. As cells grew respiring galactose, dissolved O_2_ remained below 0.25% (Supplementary Fig. 2). Importantly, under those growth conditions, *S. cerevisiae* cell-free extracts exhibited aconitase activity (405±15 nmol *cis*-aconitate·per minute·per millilitre; mean±s.d.), which is known to be sensitive to reactive oxygen species^24^. *In vivo* half-life of the *A. vinelandii* NifH [4Fe–4S] cluster is ∼5 min under standard growth conditions, comparable to the O_2_-sensitive [4Fe–4S] cluster of *E. coli* aconitase^25^. Mitochondrial his-tagged NifH (yNifHmit) was purified from *S. cerevisiae* GF2 cells co-expressing mitochondrial NifM (yNifMmit) by anaerobic Co^2+^ affinity chromatography inside a glove box (Fig. 2a). A cytosolic version (lacking the mitochondrial leader sequence; Supplementary Fig. 1) of the his-tagged NifH (yNifHcyt) was also produced and purified from *S. cerevisiae* GF9 cells co-expressing yNifMcyt (Fig. 2b). In contrast to the colourless yNifHcyt, pure yNifHmit exhibited the characteristic brown colour of nitrogenase Fe protein.

yNifHmit was capable of acting as Fe protein in the donation of electrons to the MoFe protein purified from *A. vinelandii* cells, both in the reduction of acetylene to yield ethylene and in the reduction of N_2_ to yield NH_3_ (Table 1). The Fe protein activity of yNifHmit varied among preparations, and maximum activity of 1,600 units (nmol C_2_H_4_ formed·per minute·per milligram MoFe protein) was obtained at Fe protein to MoFe protein molar ratio of 200 (Supplementary Fig. 3). Importantly, under these conditions, yNifHmit was able to support the formation of as much as 800 nmol NH_3_·per minute·per milligram MoFe protein. Co-expression of NifH with NifM was required for Fe protein activity. When mitochondrially located NifH was expressed and purified from *S. cerevisiae* GF12 cells, which lack *nifM*, yNifHmit preparations were colourless and showed no Fe protein activity (Fig. 2c and Table 1). Fe protein activity of purified yNifHmit was independent of the aeration rate of the culture in the range of 0.1 to 1 litre air·per minute·per litre of culture.

In contrast, yNifHcyt exhibited no Fe protein activity, in agreement with previous data of expression of inactive NifH in *S. cerevisiae*^26^. To investigate whether O_2_ damage was inactivating yNifHcyt at the cytosol, aerated *S. cerevisiae* GF9 cultures expressing NifH were sparged with N_2_ during 4 h before collecting the cells. The yNifHcyt purified from N_2_-treated *S. cerevisiae* cultures (Fig. 2b) exhibited very low levels of Fe protein activity (Table 1) consistent with the *in vivo* repair of a small fraction of NifH metal clusters during the anoxic period. Altogether, these data show that mitochondrial targeting is necessary to obtain active Fe protein under aerobic growth conditions, probably by offering protection against O_2_ damage.

### NifU and NifS requirement for Fe protein activity in yeast

Mitochondria carry the essential biosynthetic machinery for the assembly of Fe–S clusters^21^. Mitochondrial Isu1/Isu2 and Nfs1 are homologues of bacterial housekeeping IscU and IscS^27^ proteins involved in general Fe–S cluster assembly and the Nif-specific NifU and NifS. It was interesting to observe that, similar to the case in diazotrophic organisms^27^, NifU and NifS were not absolutely needed to produce active Fe protein in mitochondria. The fact that yNifHmit was purified in active form demonstrates the ability of the yeast machinery to load [4Fe–4S] clusters into apo-NifH.

In an attempt to enhance the Fe protein activity of yNifHmit, we generated a *S. cerevisiae* strain (GF8) expressing mitochondria-targeted and affinity-tagged NifH, NifM, NifU and NifS. yNifHmit was then purified from GF8 cell-free extracts, and Fe protein activity was determined after addition of pure *A. vinelandii* MoFe protein. Table 1 shows that co-expression of NifH and NifM with NifU and NifS did not improve mitochondrial Fe protein activity. In addition, *S. cerevisiae* GF11 strain expressing non-tagged NifU and NifS along with tagged NifH and NifM was generated to facilitate yNifHmit purification by preventing contamination with yNifUmit (Supplementary Fig. 4 and Fig. 2d), but no improvement in Fe protein activity over yNifHmit preparations from GF8 cells was observed.

Table 1 also shows that co-expression of cytosolic versions of NifU, NifS, NifH and NifM in GF13 cells subjected to 4 h of culture under anoxic conditions rendered similar levels of active Fe protein than the corresponding mitochondrial preparations from aerated GF8 cells. This result suggests that the failure to activate yNifHcyt under anoxic conditions in the absence of NifU and NifS (see above) was due to inability of the cytosolic CIA system to provide [4Fe–4S] clusters to apo-NifH. Importantly, aerobically grown GF13 cells rendered completely inactive Fe protein.

**Figure 3:**
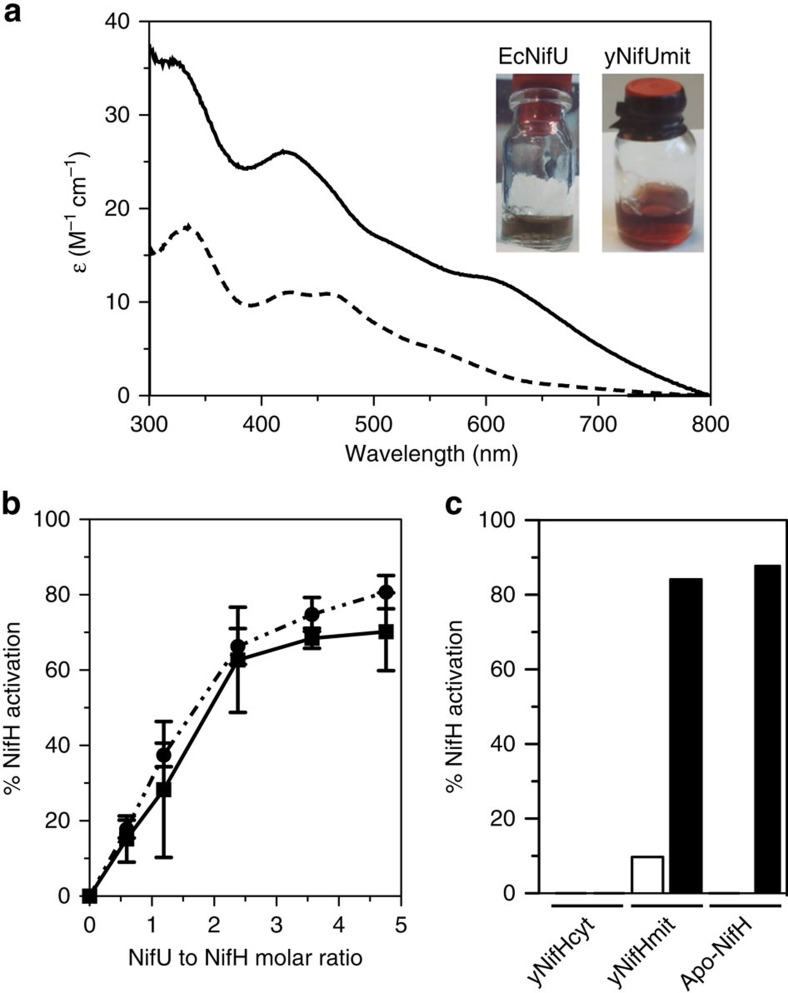
Characterization of purified yNifUmit. (**a**) Purified preparations and ultraviolet–visible spectra of yNifUmit (190 μM, broken line) and EcNifU (190 μM, solid line). (**b**) Titration of apo-NifH activation by yNifUmit (broken line; *n*=4) or EcNifU (solid line; *n*=2). Data represent means±s.d. All assays contained 5 μM apo-NifH (162 μg) in the Fe-S cluster reconstitution phase. Fully active Fe protein supported MoFe protein specific activity of 2,200 nmol ethylene·per minute·per milligram MoFe protein (corresponding to 100% activity). (**c**) Activation assays of yNifHmit (40 μg) and yNifHcyt (80 μg) with a 5-fold molar excess of yNifUmit. The yNifHmit and yNifHcyt proteins were purified from aerated cultures (1 litre air·per minute per litre of culture) of GF2 and GF9 strains, respectively. *In vitro* generated *A. vinelandii* apo-NifH (80 μg) was used as control of activation reactions. MoFe protein-specific activity supported by fully active Fe protein under same assay conditions was 1,480 nmol ethylene· per minute per milligram MoFe protein (corresponding to 100% activity). Empty bar represents the Fe protein activity of purified yNifHmit before the yNifUmit-dependent activation assay. Non-activated *A. vinelandii* apo-NifH and yNifHcyt lacked Fe protein activity. yNifUmit was unable to endow yNifHcyt with Fe protein activity.

To determine whether mitochondria-targeted NifU was active *in vitro*, his-tagged yNifUmit was purified from *S. cerevisiae* GF6 strain carrying codon-optimized synthetic versions of the *A. vinelandii nifU* and *nifS* genes (Fig. 2e) and its properties compared with those of NifU purified from a recombinant *E. coli* strain carrying *A. vinelandii nifU* and *nifS* (EcNifU). Pure yNifUmit preparations were brown, contained two Fe atoms per dimer and exhibited ultraviolet–visible spectra characteristic of [2Fe–2S] proteins^28^, with ɛ420 values consistent with the presence of a single [2Fe–2S] cluster per yNifUmit dimer (Fig. 3a). In contrast, EcNifU contained eight Fe atoms per dimer and its ultraviolet–visible spectrum was typical of [4Fe–4S] proteins. It is known that *A. vinelandii* NifU carries one permanent [2Fe–2S] cluster and one or two labile [4Fe–4S] clusters to be donated to target proteins, which are normally lost during purification^29^.

Activity of pure NifU preparations was tested by its ability to reconstitute [Fe–S] clusters into *A. vinelandii* apo-NifH in reactions containing Na_2_S as the source of sulfide and Fe(NH_4_)_2_(SO_4_)_2_ as the source of ferrous iron. Both EcNifU and yNifUmit were able to reconstitute apo-NifH with high efficiency, generating similar amounts of active Fe protein (Fig. 3b). The substitution of cysteine and *A. vinelandii* NifS for Na_2_S in the NifU-dependent apo-NifH reconstituting reactions also rendered active Fe protein (1,800 and 1,704 nmol ethylene·per minute·per milligram MoFe protein for yNifUmit and EcNifU reconstituted preparations, respectively), demonstrating the ability of EcNifU and yNifUmit to accept S atoms from NifS.

Pure yNifUmit was also used to investigate whether *S. cerevisiae* NifH preparations could be activated by [Fe–S] cluster insertion *in vitro* (Fig. 3c). Surprisingly, while yNifHmit activity could be restored to levels as high as those of the *A. vinelandii* NifH, yNifHcyt preparations were unable to accept [Fe–S] clusters from yNifUmit, even in the presence of the thiol reductant β-mercaptoethanol, suggesting that the defect in yNifHcyt is more complex than lacking its [4Fe–4S] cluster. In addition, these results demonstrate that yNifHmit is produced in a mixture of active and NifU-activatable forms.

In conclusion, this work shows that yeast mitochondria are capable of accumulating active NifU and Fe protein despite their extreme O_2_ sensitivity. The Fe protein is particularly relevant because it is one of the two structural components of nitrogenase. It is also noteworthy that endogenous mitochondrial Fe–S cluster assembly machinery is able to provide NifU and NifH with metal clusters, at least to some extent. By breaking through the limitation imposed by O_2_ in the production of an active nitrogenase within a eukaryotic cell, this work provides enabling technology that is instrumental for the grand challenge of engineering N_2_-fixing cereals.

## Methods

### Expression of Nif proteins in *S. cerevisiae*

Inoculating cultures were prepared by growing *S. cerevisiae* at 30 °C and 200 r.p.m. in 1-litre flasks containing 500 ml of SD medium supplemented with auxotrophic requirements to an *D*_600_ of 0.6. For protein purification experiments, *S. cerevisiae* cells were cultivated in 20-litre batches of SD medium supplemented with 0.5% of yeast extract, 2.5% of bactopeptone and 100 μM of ammonium iron (III) citrate, in a 250-litre fermentor (Bioprocess Technology, Spain) until glucose was consumed, at which time 2% galactose and 8 ml of a trace element solution (13 g l^−1^ CaCl_2_, 2.5 g l^−1^ MnCl_2_, 0.5 g l^−1^ ZnSO_4_, 1.4 g l^−1^ Na_2_ MoO_4_, 1.85 g l^−1^ FeCl_3_, 1 g l^−1^ H_3_BO_4_, 0.7 g l^−1^ IK in 2 M HCl) was added to induce *nif* gene expression. Dissolved O_2_ was monitored continuously during the fermentation processes by using a Mettler Toledo InPro 6850 sensor immersed into the culture. The cells were collected under anaerobic conditions 20 h after galactose addition by using hollow fiber filtration followed by centrifugation at 4 °C and 4,000 r.c.f. for 5 min. The recovered cell paste was frozen and stored into liquid N_2_ until used.

### yNifH purification

*S. cerevisiae* cells (strains GF2, GF8, GF9, GF11, GF12 and GF13) were resuspended in anaerobic lysis buffer containing 100 mM Tris-HCl pH 8.0, 100 mM NaCl, 1 mM phenylmethylsulfonyl fluoride (PMSF), 1 μg ml^−1^ leupeptin, 2 mM sodium dithionite (DTH), 5 μg ml^−1^ DNaseI, and 1 mg g^−1^ cell of Zymolyase-20T (Nacalai Tesque). The cells were lysed in a French Press cell at 25,000 lb per square inch. Cell-free extracts were obtained after removing cell debris by centrifugation at 73,000*g* for 1 h at 4 °C under anaerobic conditions. The yNifH protein was purified by Co^2+^ affinity chromatography under anaerobic conditions (<0.1 p.p.m. O_2_) using an AKTA Prime FPLC system (GE Healthcare) inside a glovebox (MBraun). In a typical purification, cell-free extract from 150 g of cell paste was loaded at 2 ml min^−1^ onto a column filled with 20 ml TALON resin (Clontech) equilibrated with lysis buffer, washed with 10 column volumes of washing buffer (100 mM Tris-HCl pH 8.0, 250 mM NaCl) and eluted with four column volumes of elution buffer (100 mM Tris-HCl pH 8.0, 250 mM NaCl, 250 mM imidazole). Elution fractions were concentrated using a Vivaspin 500 concentrator (Sartorius) with cutoff pore size of 30 kDa, and then desalted in PD10 or HiPrep 26/10 columns (GE Healthcare) equilibrated with 50 mM Tris-HCl pH 8, and 300 mM NaCl. Purified yNifHmit or yNifHcyt preparations were frozen as droplets in liquid N_2_. When purifying from GF8 cells, yNifHmit preparations contained large amounts of yNifUmit.

### yNifU purification

*S. cerevisiae* GF6 cells were resuspended in anaerobic Buffer B (100 mM Tris-HCl pH 7.9, 500 mM NaCl, 10% glycerol, 10 mM MgCl_2_) supplemented with 0.2 mM PMSF, 1 μg ml^−1^ leupeptin, 5 μg ml^−1^ DNaseI, and 0.5 mg g^−1^ cell of Zymolyase-20 T. The cells were lysed in an Emulsiflex-C5 homogenizer (Avestin Inc.) at 25,000 lb per square inch. The cell-free extracts were obtained after removing debris by centrifugation at 100,000*g* for 1 h at 4 °C under anaerobic conditions. The cell-free extracts were supplemented with 0.5 mM of L-cysteine, 2 mM of Fe(NH_4_)_2_(SO_4_)_2_ and 2 mM of β-mercaptoethanol, and incubated for 1 h at room temperature. The yNifU protein was purified by Ni^2+^affinity chromatography under anaerobic conditions (<0.1 p.p.m. O_2_) using an AKTA Prime FPLC system (GE Healthcare) inside a glovebox (MBraun). In a typical purification, the cell-free extract from 200 g of cell paste was loaded at 2 ml min^−1^ onto a column filled with 5 ml IMAC resin (GE Healthcare) equilibrated with Buffer B and washed with three successive washes of Buffer B supplemented with 15 mM imidazole, 30 mM, and 45 mM imidazole, respectively. Bound protein was eluted with elution buffer containing 50 mM Tris-HCl pH 7.9, 150 mM NaCl, 10% glycerol and 250 mM imidazole. The eluted fractions were concentrated using a 30-kDa cutoff pore Centricon 500 (Millipore) and then desalted in PD10 columns equilibrated with 50 mM Tris-HCl pH 7.9, 150 mM NaCl and 10% glycerol. Purified yNifU preparations were frozen as droplets in liquid N_2_.

### Fe protein activity determination

Fe protein activity of yNifH preparations purified from *S. cerevisiae* cells was routinely analysed by the acetylene reduction assay after addition of excess MoFe protein and ATP-regenerating mixture (1.23 mM ATP, 18 mM phosphocreatine, 2.2 mM MgCl_2_, 3 mM DTH and 40 μg of creatine phosphokinase)^10^. Positive control reactions were carried out with pure preparations of *A. vinelandii* Fe protein and MoFe protein^8^. To determine NH_3_-producing activity of MoFe protein with yNifH, argon gas was evacuated from reaction vials and replaced by N_2_ at 1 atm. The reactions were carried out at 30 °C for 60 min and then stopped by the addition of 50 mM EDTA. The NH_3_ produced was determined by liquid chromatographic fluorescence according to ref. 30 with modifications. Two hundred microlitres of each reaction mixture were withdrawn, mixed with 1 ml of fluoraldehyde and incubated in the dark for 60 min. Five hundred microlitres of these mixtures were subjected to HPLC separation (Agilent 1200 HPLC), and the ammonia-containing fractions were collected in multiwell black plates and their fluorescence quantified with a Tecan/ GENios Pro fluorescence detector.

### *In vitro* apo-NifH reconstitution assays

Apo-NifH from *A. vinelandii* was prepared *in vitro* according to ref. 31. The presence of apo-NifH in pure yNifH preparations was determined by the *in vitro* reconstitution of its [4Fe–4S] cluster according to ref. 20 with the following modifications. Each [4Fe–4S] cluster assembly mixture included 25 mM Tris-HCl pH 7.4, 3 mM β-mercaptoethanol, 50 μM NifU (either yNifU or EcNifU), 1 mM Fe(NH_4_)_2_(SO_4_)_2_ and either 0.5 mM of Na_2_S or 20 μM EcNifS plus 0.5 mM L-cysteine. The reaction mixtures were incubated for 3 h at 12 °C to allow the formation of [Fe–S] clusters onto NifU. Assays lacking NifU were carried out as control reactions. Apo-NifH or yNifH samples were then added to the [4Fe–4S] cluster assembly reaction mixture. In NifU titration experiments, 162 μg apo-NifH were assayed in combination with different volumes of the [4Fe–4S] cluster assembly reaction to obtain NifU/NifH molar ratios of 0.6, 1.2, 1.8, 2.4, 3.6 and 4.8. To establish the 100% Fe protein activity level under the same reaction conditions, control reactions lacking apo-NifH and containing 162 μg NifH were carried out. In yNifHmit and yNifHcyt reconstitution assays, 40–80 μg yNifH and a NifU to NifH molar ratio of 5 were used. In all the cases, acetylene reduction assays were conducted immediately after addition of apo-NifH to the [4Fe–4S] cluster assembly reactions by adding 30–60 μg of pure MoFe protein, 600 μl of an ATP mix and 7% acetylene, followed by incubation during 15 min at 30 °C in a rotary shaker. The reactions were stopped by adding 0.1 ml of 8 N NaOH and the ethylene formed were measured with a Shimadzu model GC2014 gas chromatograph equipped with a Porapak N^10^.

### Aconitase activity and protein methods

Aconitase activity was determined in cell-free extracts according to ref. 32. Reaction mixtures containing 0.1 ml of *S. cerevisiae* GF2 cell-free extract, 0.9 ml of anaerobic lysis buffer were incubated at room temperature. Formation of *cis-*aconitate was measured after the addition of 2 mM DL-isocitrate by following the increase in absorbance at 240 nm. Protein concentration was determined by bicinchoninic acid method (Pierce), with bovine serum albumin as the standard^33^. Colorimetric Fe determination was performed by the α,α′ bipyridyl method^34^. Protein samples (0.2 ml) were incubated with 0.1 ml of HCl 0.1 N for 60 min at 100 °C, cooled down to room temperature, mixed with 0.7 ml of a solution containing 200 mM hydroxylamine and 1.72 mM α,α′ bipyridyl, and incubated for 30 min at room temperature. The resulting absorbance at 520 nm was determined in a ultraviolet–visible spectrophotometer and referenced to calibration curves obtained with known amounts of Fe. SDS–PAGE was performed by standard methods. Immunoblot analyses were carried out with antibodies raised against the *A. vinelandii* NifH (1:5,000 dilution), NifU (1:3,000 dilution), and NifS (1:250 dilution) proteins, the Flag tag (1:2,500 dilution, Sigma Aldrich catalogue #F7425) or the *S. cerevisiae* Yah1 protein (1:500 dilution; antibody kindly donated by Rolland Lill, Marburg University). Secondary anti-rabbit antibodies were used at 1:10,000 dilution (Sigma Aldrich catalogue #A3687). Full immunoblot scans are shown in Supplementary Fig. 5.

### Confocal fluorescence microscopy

*S. cerevisiae* GF3, GF4, GF5 and GF7 strains were grown at 30 °C in 1-litre flasks containing 500 ml of SD medium supplemented with auxotrophic requirements to an *D*_600_ of 0.6. The cells were then collected by centrifugation, washed with SD medium lacking glucose, resuspended in SD medium supplemented with 2% galactose to induce NifH and NifM expression, and incubated under the same conditions for 24 h. The induced cells were collected at 4 °C by centrifugation at 4,500 r.c.f. for 5 min and then resuspended in yeast suspension buffer containing 50 mM Tris-HCl pH 7.5, 5 mM EDTA, 10% glycerol and 1 mM PMSF. Whole-cell fluorescence was analysed by using a confocal microscope Leica TCS SP8 equipped with a PL APO 40 × /1,1 water immersion objective. Excitation beam splitters TD 488/561/633 were used to capture detailed localization images of yEGFP-NifH, NifM-mKO, yEGFP-NifS and NifU-mKO fusion proteins. In parallel, galactose-induced cells were treated with 200 nM MitoTracker Deep Red (Invitrogen) to stain for mitochondria according to the manufacturer's instructions.

### Isolation and analysis of mitochondrial preparations

*S. cerevisiae* GF8 strain was grown at 30 °C in 2-litre flasks containing 1 litre of SD medium supplemented with auxotrophic requirements to an *D*_600_ of 0.6. The cells were then collected by centrifugation, washed with SD medium lacking glucose, resuspended in SD medium supplemented with 2% galactose to induce NifH, NifM, NifU and NifS expression, and incubated under the same conditions for 24 h. The procedure for mitochondria isolation has been described^35^. Ten grams of galactose-induced *S. cerevisiae* cells were collected at 4 °C by centrifugation at 3,000 r.c.f. for 5 min, resuspended in 40 ml of SP buffer (1.2 M sorbitol, 20 mM potassium phosphate pH 7.4) and incubated with Zymolyase-20T (2 mg g^−1^ cells) during 30 min at 30 °C under gentle shaking to obtain spheroplasts. All subsequent operations were conducted at 0 °C. The spheroplasts were collected by centrifugation at 800 r.c.f. for 5 min, washed and resuspended in SHP buffer (1.2 M sorbitol, 40 mM HEPES-KOH, pH 7.4, 1 mM PMSF) and homogenized in a 50-ml glass homogenizer. The cell debris was removed by centrifuging twice at 800 r.c.f. for 5 min. The mitochondria in the supernatant fraction were pelleted by centrifugation at 17,000 r.c.f. for 10 min, resuspended in 20 ml of SH buffer (0.6 M sorbitol, 20 mM HEPES-KOH, pH 7.4), subjected again to differential centrifugation at 800 and 17,000 r.c.f. and finally resuspended in 0.5 ml of SH buffer. Mitochondrial preparations were stored at −80 °C until used. The assay for integrity of mitochondria involves their treatment with proteinase K (5 ng μl^−1^ mitochondria) in the absence or presence of 2 mM CaCl_2_ and 1% Triton X-100 for 30 min at 30 °C and the analysis of the degree of proteolytic protection by SDS–PAGE and immunoblot. The Yah1 ferredoxin, is used as reference of protein present in the mitochondrial matrix.

## Additional information

**How to cite this article:** López-Torrejón, G. *et al.* Expression of a functional oxygen-labile nitrogenase component in the mitochondrial matrix of aerobically grown yeast. *Nat. Commun.* 7:11426 doi: 10.1038/ncomms11426 (2016).

## Supplementary Material

Supplementary Information Supplementary Figures 1-5, Supplementary Table 1, Supplementary Methods and Supplementary References.

## Figures and Tables

**Table 1:** MoFe protein activity supported by yNifHmit and yNifHcyt purified preparations.

**Fe protein**	**Other co-expressed Nif proteins**	**C**_**2**_**H**_**2**_ reduction activity*	**N**_**2**_ **fixation activity**†	**Fe protein to MoFe protein molar ratio**
*Mitochondrial*				
yNifHmit	NifM	404±62	ND	20
yNifHmit	NifM	1,600‡§	826±60	200
yNifHmit	None	<0.01	ND	≥400
yNifHmit||	NifM, NifU, NifS	430±45§	511±10	30
				
*Cytosolic*				
yNifHcyt (air)	NifM	<0.01	ND	≥400
yNifHcyt (air)	NifM, NifU, NifS	<0.01	ND	≥400
yNifHcyt (N_2_)	NifM	102±2	ND	≥400
yNifHcyt (N_2_)	NifM, NifU, NifS	404±20	ND	30
NifH¶		1,652±23	849±25	40

ND, not determined.

^*^Defined as nmol of C_2_H_4_ formed· per minute per milligram MoFe protein (mean±s.d., *n*=3).

^†^Defined as nmol of NH_3_ formed·per minute per milligram MoFe protein (mean±s.d., *n*=2).

^‡^Single determination.

^§^See activity titrations in Supplementary Fig. 3.

^||^Purified from GF8 cells.

^¶^Purified from *A. vinelandii*.
