# Supplementary material for: Expression of a functional oxygen-labile nitrogenase component in the mitochondrial matrix of aerobically grown yeast
Source: Nat Commun. 2016 Apr 29;7:11426. doi: 10.1038/ncomms11426 (PMC4855529; doi:10.1038/ncomms11426)
Supplement: Supplementary Information — Supplementary Figures 1-5, Supplementary Table 1, Supplementary Methods and Supplementary References. [file ncomms11426-s1.pdf]

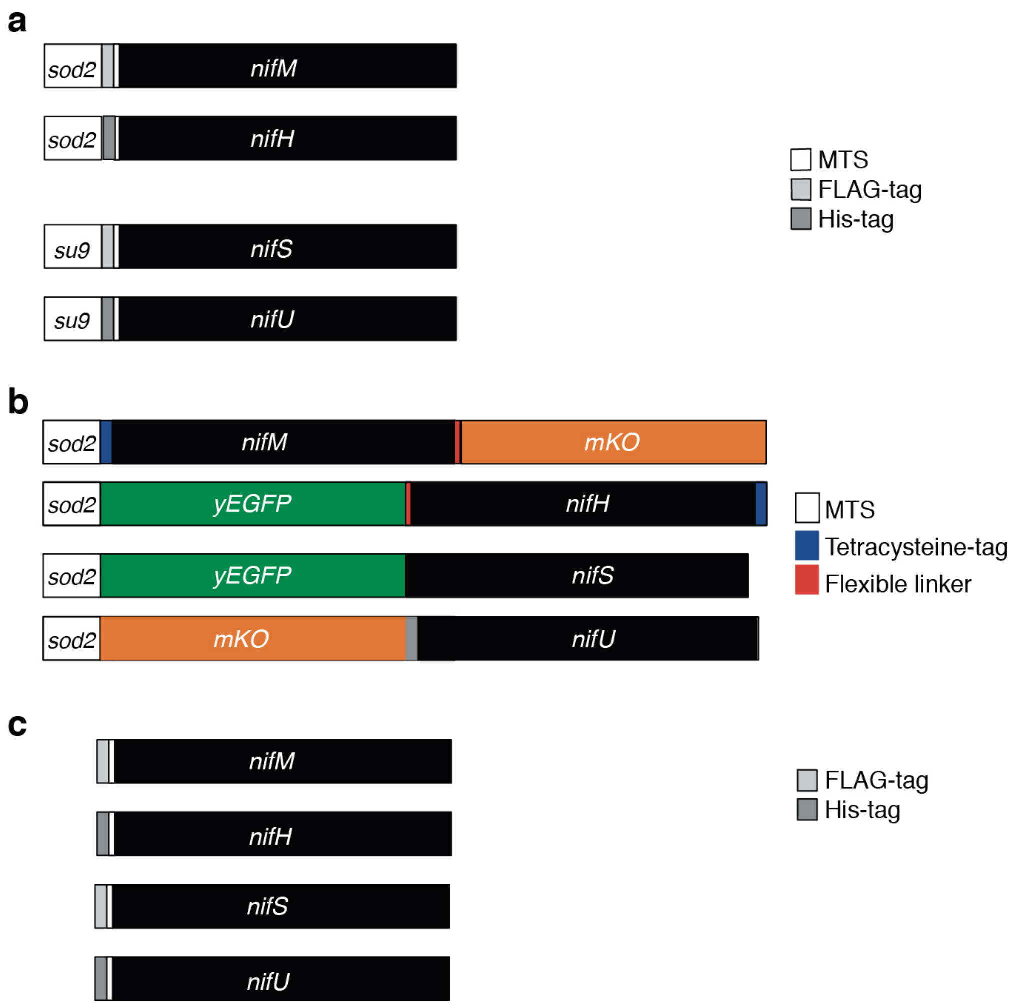

Supplementary Figure 1. Schematic representation of synthetic *nif* genes with codon optimization for *S. cerevisiae*. (**a**) Mitochondrial versions of *sod2-flag-nifM, sod2-*7x*his-nifH, Su9-flag-nifS,* and *Su9-10xhis-nifU* genes. (**b**) Mitochondrial versions of *sod2-nifM-mKO, sod2-yEGFP-nifH*, *sod2-yEGFP-nifS*, and *sod2-mKO-nifU* genes fused to fluorescent protein encoding genes. Fusion proteins include short amino acid sequences that serve as flexible linkers (shown in red) and tetracysteine motifs (shown in blue) that could be used as alternative to fluorescent proteins in intracellular location experiments. (**c**) Cytoplasmic versions of *flag-nifM*, 10x*his-nifH*, *flag-nifS*, and *10xhis-nifU*. Genes and tags are not shown to scale.

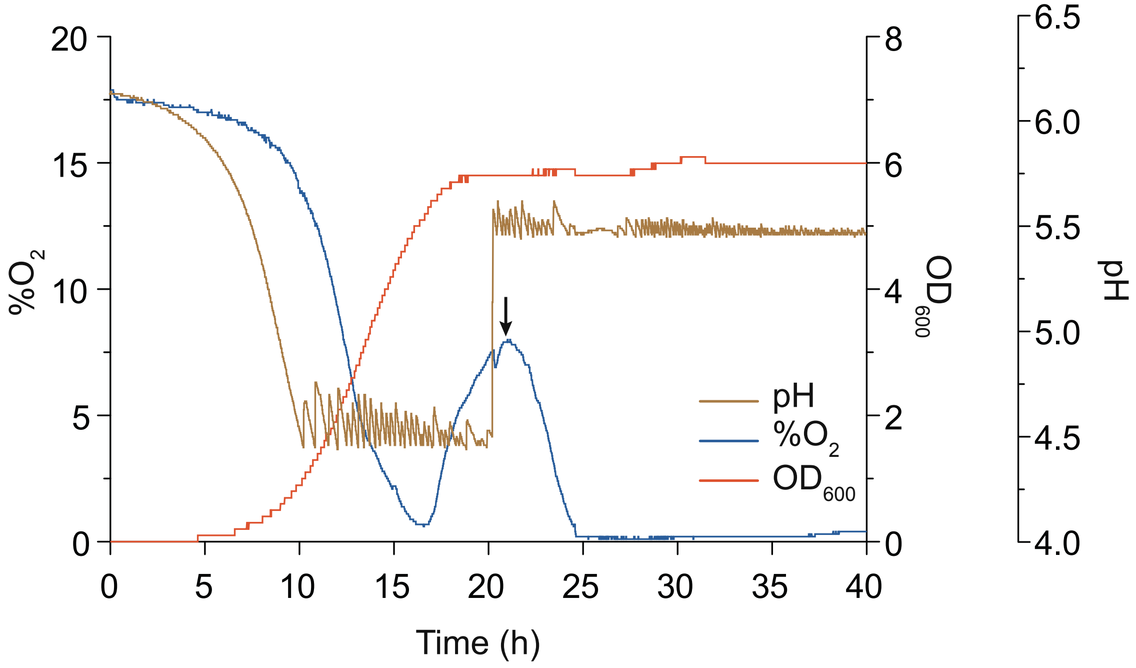

Supplementary Figure 2. Typical batch fermentation process of *S. cerevisiae* W303-1a GF2 strain expressing yNifHmit and yNifMmit. Culture turbidity (red), pH (brown), and pO_2 (blue) were continuously followed during the process by using immersed probes. Arrow indicates addition time of trace-metal and 2% galactose solutions. During the fermentation pH was automatically controlled by alkali addition. However, the pH set point was increased manually to 5.5 at 20 h. The increase in O_2 tension after 16 h coincides with exhaustion of glucose. Galactose was present at the culture medium at the time of cell harvesting.

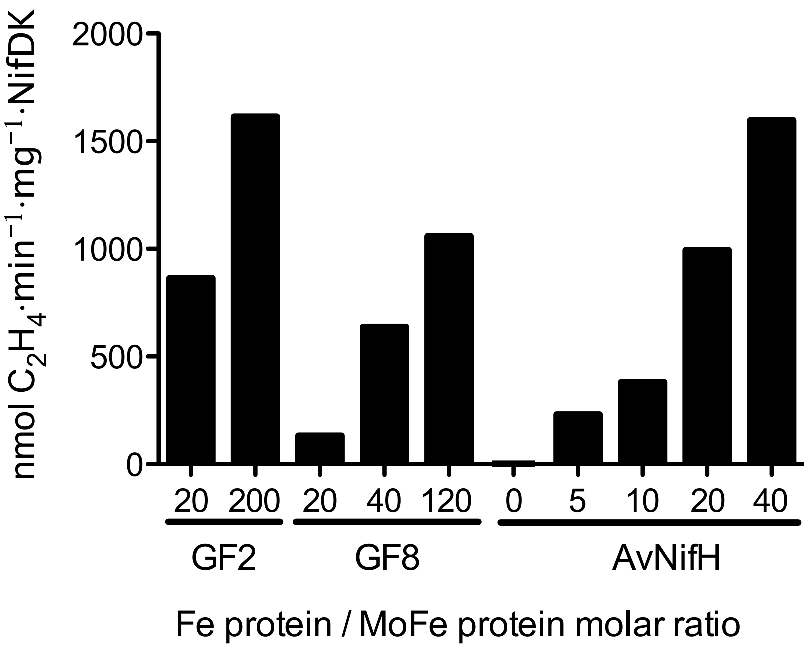

Supplementary Figure 3. Titration of MoFe protein with purified yNifHmit from *S. cerevisiae* GF2 and GF8 cells. Strain GF2 co-expresses mitochondria-targeted NifH and NifM proteins; strain GF8 co-expresses mitochondria-targeted NifH, NifM, NifU, and NifS proteins. Activities were determined by the acetylene reduction assay. Titration of *A. vinelandii* MoFe protein with the *A. vinelandii* Fe protein (AvNifH) was carried out as control.

a.MITOCHONDRIAL VERSIONS

GAL10p::*mlsSOD2-flag-nifM* in pESC-His

atcgat*atgttcgctaagaccgctgccgctaacttgactaagaaaggtggtttatcattg*
M F A K T A A A N L T K K G G L S L
ttgtccatgttcgctaagaccgccgccgctaatttaaccaaaaagggtggttgtccttg
L S M F A K T A A A N L T K K G G L S L
*ttgagt*gattacaaggatgacgatgacaagattgcatccgaaagattagccgatggtgac
L S **D Y K D D D D K I** A S E R L A D G D
agtagatattacttgttaaaagttgcccatgaacaatttggttgcgctcctggtgaatta
S R Y Y L L K V A H E Q F G C A P G E L
tccgaagaacaattgcaacaagctgatagaattataggtagacaaagacacatagaagat
S E E Q L Q Q A D R I M G R Q R H M E D
gcagttttaagatctccagacgccataggtgttgtcatcccaccttcacaattggaagaa
A V L R S P D A M G V V I P P S Q L E E
gcatgggcccatattgcatctagatacgaatcacctgaagctttgcaacaagcattagat
A W A H I A S R Y E S P E A L Q Q A L D
gctcaagcattggacgctgctggtatgagagccatgttggctagagaattaagagtcgaa
A Q A L D A A G M R A M L A R E L R V E
gctgtattagattgtgtctgcgcaggtttgccagaaatatccgatactgacgttagtttg
A V L D C V C A G L P E M S D T D V S L
tactacttcaaccatgctgaacaattcaaagtaccagcccaacataaggctagacacatt
Y Y F N H A E Q F K V P A Q H K A R H I
ttggttaccataaatgaagactttcctgaaaacactagagaagccgctagaactagaatc
L V T M N E D F P E N T R E A A R T R I
gaaacaatcttgaagagattgagaggtaaaccagaaagattcgcagaacaagccatgaaa
E T I L K R L R G K P E R F A E Q A M K
cactctgaatgtcctacagctatgcaaggtggtttgttaggtgaagtagttccaggtacc
H S E C P T A M Q G G L L G E V V P G T
ttgtatcctgaattagatgcttgcttgtttcaaatggcacgtggtgaattatctccagtt
L Y P E L D A C L F Q M A R G E L S P V
ttggaatcacctatcggtttccatgttttatactgtgaatctgtctcaccagccagacaa
L E S P I G F H V L Y C E S V S P A R Q
ttgacattggaagaaatcttgcctagattgagagatagattgcaattgagacaaagaaag
L T L E E I L P R L R D R L Q L R Q R K
gcatatcaaagaaaatggttagaatccttattacaacaaaacgccaccttagaaaactta
A Y Q R K W L E S L L Q Q N A T L E N L
gcacacggttaagagctc
A H G *

GAL1p::*mlsSOD2-his*$_7$*-nifH* in pESC-His

```
gtcgacatgtttgctaagaccgccgccgctaacttgactaagaagggtggtttgagttta
      M  F  A  K  T  A  A  A  N  L  T  K  K  G  G  L  S  L
ttatctatgtttgctaagacagccgccgctaacttaaccaaaaagggtggtttgtccttg
 L  S  M  F  A  K  T  A  A  A  N  L  T  K  K  G  G  L  S  L
ttaagtcatcaccatcaccatcaccatgctatgagacaatgtgcaatctatggtaaaggt
 L  S  H  H  H  H  H  H  H  A  M  R  Q  C  A  I  Y  G  K  G
ggtatcggtaaatctactacaacccaaaatttggttgctgcattagctgaaatgggtaaa
 G  I  G  K  S  T  T  T  Q  N  L  V  A  A  L  A  E  M  G  K
aaggttatgattgtcggttgcgatccaaaagctgactctactagattgatcttacactca
 K  V  M  I  V  G  C  D  P  K  A  D  S  T  R  L  I  L  H  S
aaggcacaaaacactattatggaaatggccgctgaagccggtacagtagaagatttggaa
 K  A  Q  N  T  I  M  E  M  A  A  E  A  G  T  V  E  D  L  E
ttagaagacgttttgaaagcaggttacggtggtgtaaagtgtgttgaatcaggtggtcca
 L  E  D  V  L  K  A  G  Y  G  G  V  K  C  V  E  S  G  G  P
gaacctggtgttggttgcgccggtagaggtgtcataacagctattaatttcttggaagaa
 E  P  G  V  G  C  A  G  R  G  V  M  T  A  I  N  F  L  E  E
gaaggtgcttacgaagatgacttagatttcgtcttctacgatgtattgggtgacgttgtc
 E  G  A  Y  E  D  D  L  D  F  V  F  Y  D  V  L  G  D  V  V
tgtggtggtttcgcaatgcctattagagaaaataaggcccaagaaatctatatagtttgc
 C  G  G  F  A  M  P  I  R  E  N  K  A  Q  E  I  Y  M  V  C
tccggtgaaatgatggcaatgtacgcagccaacaacattagtaagggtatcgtcaagtac
 S  G  E  M  M  A  M  Y  A  A  N  N  I  S  K  G  I  V  K  Y
gctaactctggttcagttagattgggtggtttgatctgtaattctagaaacaccgataga
 A  N  S  G  S  V  R  L  G  G  L  I  C  N  S  R  N  T  D  R
gaagacgaattgatcatcgctttggcaaataagttgggtactcaaatgatccatttcgtt
 E  D  E  L  I  I  A  L  A  N  K  L  G  T  Q  M  I  H  F  V
ccaagagataacgtagttcaaagagctgaaataagaagaatgacagttatagaatacgat
 P  R  D  N  V  V  Q  R  A  E  M  R  R  M  T  V  M  E  Y  D
cctaaagcaaagcaagccgacgaatacagagccttagctagaaaggtcgtagataataag
 P  K  A  K  Q  A  D  E  Y  R  A  L  A  R  K  V  V  D  N  K
ttgttagttatcccaaaccctattaccatggatgaattggaagaattgttaatggaattt
 L  L  V  I  P  N  P  I  T  M  D  E  L  E  E  L  L  M  E  F
ggtattatggaagtagaagatgaatcaatcgtcggtaaaactgctgaagaagtctgactc
 G  I  M  E  V  E  D  E  S  I  V  G  K  T  A  E  E  V  *
gag
```

GAL10p::*mlsSu9-flag-nifS* in pESC-Ura

gaattc*atggcctccactcgtgtcctcgcctctcgcctggcctcccagatggctgcttcc*

M A S T R V T A S R T A S Q M A A S

gccaaggttgcccgccctgctgtccgcgttgctcaggtcagcaagcgcaccatccagact

A K V A R P A V R V A Q V S K R T I Q T

ggctccccctccagaccctcaagcgcacccagatgacctccatcgtcaacgccaccacc

G S P T Q T T K R T Q M T S I V N A T T

*cgccaggctttccagaagcgcgcctactcttcc*agcggccgcgattataaagatgacgat

R Q A F Q K R ***A Y*** *S S* S G R **D Y K D D D**

gacaagattgtcgacgccgacgtttacttggataataacgctactacaagagtcgatgac

D K I V D A D V Y L D N N A T T R V D D

gaaatagtacaagctatgttgccattttcacagaacaattcggtaacccttccagtttg

E M V Q A M L P F F T E Q F G N P S S L

cattccttcggtaaccaagttggtatggccttgaagaaagctagacaatctgtccaaaaa

H S F G N Q V G M A L K K A R Q S V Q K

ttgttaggtgcagaacacgattccgaaatcgtttttaccagttgtggtactgaatctgac

L L G A E H D S E I V F T S C G T E S D

tcaaccgccatttgtctgccttaaaagctcaaccagaaagaaagactgtcataaccact

S T A I L S A L K A Q P E R K T V M T T

gttgtcgaacatcctgcagtattgtctttatgcgattatttggcctcagaaggttacact

V V E H P A V L S L C D Y L A S E G Y T

gttcataagttaccagtcgataaaaagggtagattggacttagaacactatgcttccttg

V H K L P V D K K G R L D L E H Y A S L

ttaacagatgacgtagctgtagttagtgttatgtgggcaaataacgaaactggtacattg

L T D D V A V V S V M W A N N E T G T L

tttccaattgaagaaatggcaagattagccgatgacgctggtataatgttccatactgat

F P I E E M A R L A D D A G M M F H T D

gcagtacaagccgttggtaaagtccctatagacttgaagaactcgtcaatccacatgttg

A V Q A V G K V P M D L K N S S I H M L

tccttaagtggtcataaattgcacgctccaaagggtgttggtgtcttgtacttaagaaga

S L S G H K L H A P K G V G V L Y L R R

ggtacaagattcagacctttgttaagaggtggtcatcaagaaagaggtagaagagccggt

G T R F R P L L R G G H Q E R G R R A G

actgaaaatgctgcatctattataggtttgggtgttgccgctgaaagagctttacaattc

T E N A A S I M G L G V A A E R A L Q F

atggaacatgaaaacactgaagttaagagattgcgtgataagttagaagcaggtattttg

M E H E N T E V K R L R D K L E A G I L

gccgtcgtaccacacgcatttgttactggtgacccagacaatagattacctaacacagct

A V V P H A F V T G D P D N R L P N T A

aacatcgcattcgaatacatcgaaggtgaagctatcttgttgttgttgaacaaagttggt

N I A F E Y I E G E A I L L L L N K V G

atagcagcctccagtggttctgcttgtacatctggttcattggaaccatcacatgttatg

M A A S S G S A C T S G S L E P S H V M

agagcaatggatattccttatacagctgcacacggtactgttagattttctttgagtaga

R A M D I P Y T A A H G T V R F S L S R

tacacaaccgaagaagaaattgatagagtcattagagaagtaccacctattgttgctcaa

Y T T E E E I D R V I R E V P P I V A Q

ttgagaaaattgtctccttactggtcaggtaatggtcctgttgaagaccctggtaaagcc

L R K L S P Y W S G N G P V E D P G K A

tttgctcctgtctatggttgaagatct

F A P V Y G *

GAL1p::*mlsSu9-his*$_{10}$*-nifU* in pESC-Ura

ggatcc*atggcctccactcgtgtcctcgcctctcgcctggcctcccagatggctgcttcc*
M A S T R V T A S R T A S Q M A A S
gccaaggttgcccgccctgctgtccgcgttgctcaggtcagcaagcgcaccatccagact
A K V A R P A V R V A Q V S K R T I Q T
ggctccccctccagaccctcaagcgcacccagatgacctccatcgtcaacgccaccacc
G S P T Q T T K R T Q M T S I V N A T T
*cgccaggctttccagaagcgcgcctactcttcc*actagtcatcaccaccatcatcaccat
R Q A F Q K R ***A Y*** *S S* T S **H H H H H H**
caccatcacatcgattgggactactctgaaaaggttaaggaacatttctacaatccaaag
H H H I D W D Y S E K V K E H F Y N P K
aacgccggtgctgtagaaggtgcaaacgccattggtgacgttggttcattatcctgtggt
N A G A V E G A N A I G D V G S L S C G
gacgctttgagattaacattgaaagttgaccctgaaaccgatgtcatcttggacgcaggt
D A L R L T L K V D P E T D V I L D A G
tttcaaactttcggttgcggttctgctattgcatcttcatccgctttgactgaaatggtt
F Q T F G C G S A I A S S S A L T E M V
aagggtttgacattggatgaagcattgaaaatctcaaaccaagatatcgctgactatttg
K G L T L D E A L K I S N Q D I A D Y L
gatggtttgccacctgaaaagatgcattgttccgtcatgggtagagaagccttacaagct
D G L P P E K M H C S V M G R E A L Q A
gcagtagctaactacagaggtgaaaccattgaagatgaccacgaagaaggtgcattgata
A V A N Y R G E T I E D D H E E G A L M
tgtaaatgctttgccgttgatgaagttatggtcagagataccataagagcaaataagtta
C K C F A V D E V M V R D T M R A N K L
agtactgtagaagatgttactaactacacaaaagctggtggtggttgttctgcttgccat
S T V E D V T N Y T K A G G G C S A C H
gaagcaatagaaagagttttgacagaagaattggccgctagaggtgaagtattcgttgca
E A M E R V L T E E L A A R G E V F V A
gccccaattaaagccaaaaagaaagtcaaggtattggctccagaacctgccccagctcct
A P I K A K K K V K V L A P E P A P A P
gttgcagaagccccagctgcagcccctaagttgtcaaatttgcaaagaattagaagaatc
V A E A P A A A P K L S N L Q R I R R I
gaaacagtcttggctgcaataagacctaccttgcaaagagacaaaggtgacgtcgaatta
E T V L A A M R P T L Q R D K G D V E L
attgatgtagacggtaaaaatgtttacgtcaaattgaccggtgcttgtactggttgccaa
I D V D G K N V Y V K L T G A C T G C Q
atggcatccatgacattaggtggtatacaacaaagattgatcgaagaattgggtgagttc
M A S M T L G G M Q Q R L I E E L G E F
gtcaaagttatcccagtctccgctgccgcacacgcccaaatggaagtctgaaagctt
V K V I P V S A A A H A Q M E V *

b. FLUORESCENT VERSIONS

GAL10p::*mlsSOD2-*cys_4*-nifM-mKO* in pESC-His

```
gaattcatgttcgctaagactgccgccgctaacttgacaaagaaaggtggtttatcatta
      M  F  A  K  T  A  A  A  N  L  T  K  K  G  G  L  S  L
ttgagtatgttcgctaagacagccgccgctaacttaactaaaaagggtggtttgagtttg
 L  S  M  F  A  K  T  A  A  A  N  L  T  K  K  G  G  L  S  L
ttatctggttcttcaggttgttgcccaggttgttgcgcctcagaaagattagctgatggt
 L  S  G  S  S  G  C  C  P  G  C  C  A  S  E  R  L  A  D  G
gactccagatattacttgttaaaagttgcccatgaacaatttggttgtgctcctggtgaa
 D  S  R  Y  Y  L  L  K  V  A  H  E  Q  F  G  C  A  P  G  E
ttatcagaagaacaattgcaacaagcagatagaattataggtagacaaagacacatagaa
 L  S  E  E  Q  L  Q  Q  A  D  R  I  M  G  R  Q  R  H  M  E
gatgcagttttaagatcaccagacgccataggtgttgtcatcccaccttcccaattggaa
 D  A  V  L  R  S  P  D  A  M  G  V  V  I  P  P  S  Q  L  E
gaagcttgggcacatattgcttcaagatatgaatcccctgaagccttgcaacaagcttta
 E  A  W  A  H  I  A  S  R  Y  E  S  P  E  A  L  Q  Q  A  L
gatgcccaagctttggacgctgctggtatgagagcaatgttggccagagaattaagagtt
 D  A  Q  A  L  D  A  A  G  M  R  A  M  L  A  R  E  L  R  V
gaagctgtcttagattgtgtctgcgcaggtttgccagaaattagtgatacagacgtatct
 E  A  V  L  D  C  V  C  A  G  L  P  E  I  S  D  T  D  V  S
ttgtactacttcaaccatgctgaacaattcaaggtaccagcacaacataaagccagacac
 L  Y  Y  F  N  H  A  E  Q  F  K  V  P  A  Q  H  K  A  R  H
atattggttactataaatgaagattttcctgaaaacacaagagaagccgctagaaccaga
 M  L  V  T  M  N  E  D  F  P  E  N  T  R  E  A  A  R  T  R
atcgaaactatcttgaagagattgagaggtaaaccagaaagattcgctgaacaagcaatg
 I  E  T  I  L  K  R  L  R  G  K  P  E  R  F  A  E  Q  A  M
aaacactctgaatgtcctacagctatgcaaggtggtttgttaggtgaagtagttccaggt
 K  H  S  E  C  P  T  A  M  Q  G  G  L  L  G  E  V  V  P  G
accttgtatcctgaattagatgcatgcttgtttcaaatggccagaggtgaattatcacca
 T  L  Y  P  E  L  D  A  C  L  F  Q  M  A  R  G  E  L  S  P
gttttggaatcccctattggtttccatgttttatactgtgaatccgtcagtccagcaaga
 V  L  E  S  P  I  G  F  H  V  L  Y  C  E  S  V  S  P  A  R
caattgaccttggaagaaatcttgcctagattgagagatagattgcaattgagacaaaga
 Q  L  T  L  E  E  I  L  P  R  L  R  D  R  L  Q  L  R  Q  R
aaggcttaccaaagaaagtggttggaatctttgttgcaacaaaatgctaccttggaaaac
 K  A  Y  Q  R  K  W  L  E  S  L  L  Q  Q  N  A  T  L  E  N
ttagcacatggtgcagccgcggggactatggtctctgtaatcaaaccagaaatgaagatg
 L  A  H  G  A  A  A  G  T  M  V  S  V  I  K  P  E  M  K  M
agatactacatggatggttcagttaacggtcatgagtttactattgaaggtgaaggtact
 R  Y  Y  M  D  G  S  V  N  G  H  E  F  T  I  E  G  E  G  T
ggtagaccttatgaaggtcaccaagaaatgacattaagagttaccatggccaagggtggt
 G  R  P  Y  E  G  H  Q  E  M  T  L  R  V  T  M  A  K  G  G
ccaatgccttttgctttcgacttggtatctcatgttttctgttacggtcacagaccattc
 P  M  P  F  A  F  D  L  V  S  H  V  F  C  Y  G  H  R  P  F
acaaagtacccagaagaaatccctgattacttcaagcaagctttccctgaaggtttaagt
 T  K  Y  P  E  E  I  P  D  Y  F  K  Q  A  F  P  E  G  L  S
tgggaaagatctttggaatttgaagacggtggttctgcttcagtttccgcacatataagt
 W  E  R  S  L  E  F  E  D  G  G  S  A  S  V  S  A  H  M  S
ttgcgtggtaacacattctaccacaagtctaagtttaccggtgttaatttcccagcagat
 L  R  G  N  T  F  Y  H  K  S  K  F  T  G  V  N  F  P  A  D
ggtcctataatgcaaaaccaaagtgtcgactgggaaccatctactgaaaagattacagct
 G  P  M  M  Q  N  Q  S  V  D  W  E  P  S  T  E  K  I  T  A
tctgatggtgtcttgaagggtgacgtaactatgtacttgaaattagaaggtggtggtaac
 S  D  G  V  L  K  G  D  V  T  M  Y  L  K  L  E  G  G  G  N
cataagtgtcaattcaaaactacatacaaggcagccaaaaagatcttgaagatgccaggt
 H  K  C  Q  F  K  T  T  Y  K  A  A  K  K  I  L  K  M  P  G
tcacattacatttcccacagattggttagaaaaacagaaggtaatatcactgaattggtt
 S  H  Y  I  S  H  R  L  V  R  K  T  E  G  N  I  T  E  L  V
gaagatgctgtcgctcactactctatgttgccttcctaaatcgat
 E  D  A  V  A  H  Y  S  M  L  P  S  *
```

GAL1p::*mlsSOD2-gfp-nifH-cys*$_4$ in pESC-Leu

```
ggatccatgttcgctaagacagccgccgctaacttgacaaagaagggtggtttgtcattg
       M  F  A  K  T  A  A  A  N  L  T  K  K  G  G  L  S  L
ctctctatgttcgcaaagactgccgcagctaacttaactaagaaaggtggtttgtcattg
 T  S  M  F  A  K  T  A  A  A  N  L  T  K  K  G  G  L  S  L
ctcagtatgtcaaagggtgaagaattgtttactggtgttgtaccaatcttggtagaatta
 T  S  M  S  K  G  E  E  L  F  T  G  V  V  P  I  L  V  E  L
gatggtgacgtcaatggtcataaattctccgttagtggtgaaggtgaaggtgacgcaaca
 D  G  D  V  N  G  H  K  F  S  V  S  G  E  G  E  G  D  A  T
tacggtaaattgaccttgaagtttatatgtactactggtaaattgccagttccttggcca
 Y  G  K  L  T  L  K  F  M  C  T  T  G  K  L  P  V  P  W  P
acattggtaaccacttttggttatggtgttcaatgcttcgccagataccctgatcatatg
 T  L  V  T  T  F  G  Y  G  V  Q  C  F  A  R  Y  P  D  H  M
aaacaacacgacttttttcaagtccgctatgccagaaggttacgttcaagaaagaactatt
 K  Q  H  D  F  F  K  S  A  M  P  E  G  Y  V  Q  E  R  T  I
ttctttaaggatgacggtaactacaagaccagagctgaagtaaagttcgaaggtgacact
 F  F  K  D  D  G  N  Y  K  T  R  A  E  V  K  F  E  G  D  T
ttggtcaacagaatcgaattgaagggtatcgatttcaaggaagacggtaacatattgggt
 L  V  N  R  I  E  L  K  G  I  D  F  K  E  D  G  N  M  L  G
cataagttggaatacaattacaactcacacaacgtatacatcatggctgataagcaaaag
 H  K  L  E  Y  N  Y  N  S  H  N  V  Y  I  M  A  D  K  Q  K
aatggtattaaagtcaacttcaagatcagacataacatcgaagatggttccgttcaatta
 N  G  I  K  V  N  F  K  I  R  H  N  I  E  D  G  S  V  Q  L
gcagaccactatcaacaaaatacccctattggtgacggtcctgttttgttaccagacaac
 A  D  H  Y  Q  Q  N  T  P  I  G  D  G  P  V  L  L  P  D  N
cattacttatccactcaaagtgctttgtctaaagatccaaatgaaaagagagaccatatg
 H  Y  L  S  T  Q  S  A  L  S  K  D  P  N  E  K  R  D  H  M
gtcttgttagaatttgttactgctgcaggtattacacacggtatggatgaattgtataaa
 V  L  L  E  F  V  T  A  A  G  I  T  H  G  M  D  E  L  Y  K
gccgccgcggggacaatggccatgagacaatgtgctatctacggtaaaggtggtattggt
 A  A  A  G  T  M  A  M  R  Q  C  A  I  Y  G  K  G  G  I  G
aaaagtacaaccactcaaaacttggttgccgctttagcagaaatgggtaaaaaggtcatg
 K  S  T  T  T  Q  N  L  V  A  A  L  A  E  M  G  K  K  V  M
attgttggttgcgatcctaaggccgactcaactagattgatattacactccaaagctcaa
 I  V  G  C  D  P  K  A  D  S  T  R  L  M  L  H  S  K  A  Q
aatacaatcatggaaatggcagccgaagcaggtaccgttgaagatttggaattggaagac
 N  T  I  M  E  M  A  A  E  A  G  T  V  E  D  L  E  L  E  D
gtattgaaggccggttatggtggtgtaaaatgtgtcgaatctggtggtcctgaaccaggt
 V  L  K  A  G  Y  G  G  V  K  C  V  E  S  G  G  P  E  P  G
gtcggttgcgcaggtagaggtgttataactgccatcaatttcttggaagaagaaggtgca
 V  G  C  A  G  R  G  V  M  T  A  I  N  F  L  E  E  E  G  A
tacgaagatgacttagatttcgttttctacgatgtattgggtgacgtcgtttgtggtggt
 Y  E  D  D  L  D  F  V  F  Y  D  V  L  G  D  V  V  C  G  G
tttgctatgccaattagagaaaacaaggcacaagaaatctatatagtttgcagtggtgaa
 F  A  M  P  I  R  E  N  K  A  Q  E  I  Y  M  V  C  S  G  E
atgatggctatgtacgctgcaaacaacatctctaagggtatagtcaagtacgccaattct
 M  M  A  M  Y  A  A  N  N  I  S  K  G  M  V  K  Y  A  N  S
ggttcagttagattgggtggtttgatctgtaactcaagaaacactgatagagaagacgaa
 G  S  V  R  L  G  G  L  I  C  N  S  R  N  T  D  R  E  D  E
ttgattatagccttagctaacaaattgggtacacaaatgatccatttcgtacctagagat
 L  I  M  A  L  A  N  K  L  G  T  Q  M  I  H  F  V  P  R  D
aatgtagtccaaagagctgaaatcagaagaatgacagttatcgaatatgatccaaaagct
 N  V  V  Q  R  A  E  I  R  R  M  T  V  I  E  Y  D  P  K  A
aagcaagcagacgaatacagagcattggccagaaaggttgtagataataagttgttagtt
 K  Q  A  D  E  Y  R  A  L  A  R  K  V  V  D  N  K  L  L  V
atacctaacccaatcactatggatgaattggaagaattgttgatggaattcggtatcatg
 M  P  N  P  I  T  M  D  E  L  E  E  L  L  M  E  F  G  I  M
gaagttgaagatgaatctatagtcggtaaaactgctgaagaagtaggtagtagtggttgt
 E  V  E  D  E  S  M  V  G  K  T  A  E  E  V  G  S  S  G  C
tgcccaggttgttgtgtcgac
 C  P  G  C  C
```

GAL10p::*mlsSOD2-gfp-nifS* in pESC-Ura

```
gaattcatgttcgctaagaccgctgctgctaacttaactaaaaagggtggtttgtcattg
       M  F  A  K  T  A  A  A  N  L  T  K  K  G  G  L  S  L
ttgagtatgttcgccaagaccgccgccgcaaatttgaccaaaaagggtggtttatcattg
 L  S  M  F  A  K  T  A  A  A  N  L  T  K  K  G  G  L  S  L
ttatctagcggccgcatgtcaaagggtgaagaattgtttactggtgttgtaccaatcttg
 L  S  S  G  R  M  S  K  G  E  E  L  F  T  G  V  V  P  I  L
gtagaattagatggtgacgtcaatggtcataaattctccgttagtggtgaaggtgaaggt
 V  E  L  D  G  D  V  N  G  H  K  F  S  V  S  G  E  G  E  G
gacgcaacatacggtaaattgaccttgaagtttatatgtactactggtaaattgccagtt
 D  A  T  Y  G  K  L  T  L  K  F  M  C  T  T  G  K  L  P  V
ccttggccaacattggtaaccacttttggttatggtgttcaatgcttcgccagataccct
 P  W  P  T  L  V  T  T  F  G  Y  G  V  Q  C  F  A  R  Y  P
gatcatatgaaacaacacgactttttcaagtccgctatgccagaaggttacgttcaagaa
 D  H  M  K  Q  H  D  F  F  K  S  A  M  P  E  G  Y  V  Q  E
agaactattttctttaaggatgacggtaactacaagaccagagctgaagtaaagttcgaa
 R  T  I  F  F  K  D  D  G  N  Y  K  T  R  A  E  V  K  F  E
ggtgacactttggtcaacagaatcgaattgaagggtatcgatttcaaggaagacggtaac
 G  D  T  L  V  N  R  I  E  L  K  G  I  D  F  K  E  D  G  N
atattgggtcataagttggaatacaattacaactcacacaacgtatacatcatggctgat
 M  L  G  H  K  L  E  Y  N  Y  N  S  H  N  V  Y  I  M  A  D
aagcaaaagaatggtattaaagtcaacttcaagatcagacataacatcgaagatggttcc
 K  Q  K  N  G  I  K  V  N  F  K  I  R  H  N  I  E  D  G  S
gttcaattagcagaccactatcaacaaaatacccctattggtgacggtcctgttttgtta
 V  Q  L  A  D  H  Y  Q  Q  N  T  P  I  G  D  G  P  V  L  L
ccagacaaccattacttatccactcaaagtgctttgtctaaagatccaaatgaaaagaga
 P  D  N  H  Y  L  S  T  Q  S  A  L  S  K  D  P  N  E  K  R
gaccatatggtcttgttagaatttgttactgctgcaggtattacacacggtatggatgaa
 D  H  M  V  L  L  E  F  V  T  A  A  G  I  T  H  G  M  D  E
ttgtataaagtcgacgccgacgtttacttggataataacgctactacaagagtcgatgac
 L  Y  K  V  D  A  D  V  Y  L  D  N  N  A  T  T  R  V  D  D
gaaatagtacaagctatgttgccatttttcacagaacaattcggtaacccttccagtttg
 E  M  V  Q  A  M  L  P  F  F  T  E  Q  F  G  N  P  S  S  L
cattccttcggtaaccaagttggtatggccttgaagaaagctagacaatctgtccaaaaa
 H  S  F  G  N  Q  V  G  M  A  L  K  K  A  R  Q  S  V  Q  K
ttgttaggtgcagaacacgattccgaaatcgtttttaccagttgtggtactgaatctgac
 L  L  G  A  E  H  D  S  E  I  V  F  T  S  C  G  T  E  S  D
tcaaccgccattttgtctgccttaaaagctcaaccagaaagaaagactgtcataaccact
 S  T  A  I  L  S  A  L  K  A  Q  P  E  R  K  T  V  M  T  T
gttgtcgaacatcctgcagtattgtctttatgcgattatttggcctcagaaggttacact
 V  V  E  H  P  A  V  L  S  L  C  D  Y  L  A  S  E  G  Y  T
gttcataagttaccagtcgataaaaagggtagattggacttagaacactatgcttccttg
 V  H  K  L  P  V  D  K  K  G  R  L  D  L  E  H  Y  A  S  L
ttaacagatgacgtagctgtagttagtgttatgtgggcaaataacgaaactggtacattg
 L  T  D  D  V  A  V  V  S  V  M  W  A  N  N  E  T  G  T  L
tttccaattgaagaaatggcaagattagccgatgacgctggtataatgttccatactgat
 F  P  I  E  E  M  A  R  L  A  D  D  A  G  M  M  F  H  T  D
gcagtacaagccgttggtaaagtccctatagacttgaagaactcgtcaatccacatgttg
 A  V  Q  A  V  G  K  V  P  M  D  L  K  N  S  S  I  H  M  L
tccttaagtggtcataaattgcacgctccaaagggtgttggtgtcttgtacttaagaaga
 S  L  S  G  H  K  L  H  A  P  K  G  V  G  V  L  Y  L  R  R
ggtacaagattcagacctttgttaagaggtggtcatcaagaaagaggtagaagagccggt
 G  T  R  F  R  P  L  L  R  G  G  H  Q  E  R  G  R  R  A  G
actgaaaatgctgcatctattataggtttgggtgttgccgctgaaagagctttacaattc
 T  E  N  A  A  S  I  M  G  L  G  V  A  A  E  R  A  L  Q  F
atggaacatgaaaacactgaagttaagagattgcgtgataagttagaagcaggtattttg
 M  E  H  E  N  T  E  V  K  R  L  R  D  K  L  E  A  G  I  L
gccgtcgtaccacacgcatttgttactggtgacccagacaatagattacctaacacagct
 A  V  V  P  H  A  F  V  T  G  D  P  D  N  R  L  P  N  T  A
aacatcgcattcgaatacatcgaaggtgaagctatcttgttgttgttgaacaaagttggt
```

N I A F E Y I E G E A I L L L L N K V G
atagcagcctccagtggttctgcttgtacatctggttcattggaaccatcacatgttatg
M A A S S G S A C T S G S L E P S H V M
agagcaatggatattccttatacagctgcacacggtactgttagattttctttgagtaga
R A M D I P Y T A A H G T V R F S L S R
tacacaaccgaagaagaaattgatagagtcattagagaagtaccacctattgttgctcaa
Y T T E E E I D R V I R E V P P I V A Q
ttgagaaaattgtctccttactggtcaggtaatggtcctgttgaagaccctggtaaagcc
L R K L S P Y W S G N G P V E D P G K A
tttgctcctgtctatggttgaagatct
F A P V Y G *

GAL1p:: *mlsSOD2-mKO-his$_{10}$-nifU* in pESC-Ura

```
ggatccatgttcgcaaagaccgccgccgctaacttgactaagaagggtggtttatcatta
        M  F  A  K  T  A  A  A  N  L  T  K  K  G  G  L  S  L
ttatctatgttcgcaaagaccgccgctgctaatttgactaaaaagggtggtttgagtttg
 L  S  M  F  A  K  T  A  A  A  N  L  T  K  K  G  G  L  S  L
ttaagtactagtatggtctctgtaatcaaaccagaaatgaagatgagatactacatggat
 L  S  T  S  M  V  S  V  I  K  P  E  M  K  M  R  Y  Y  M  D
ggttcagttaacggtcatgagtttactattgaaggtgaaggtactggtagaccttatgaa
 G  S  V  N  G  H  E  F  T  I  E  G  E  G  T  G  R  P  Y  E
ggtcaccaagaaatgacattaagagttaccatggccaagggtggtccaatgccttttgct
 G  H  Q  E  M  T  L  R  V  T  M  A  K  G  G  P  M  P  F  A
ttcgacttggtatctcatgttttctgttacggtcacagaccattcacaaagtacccagaa
 F  D  L  V  S  H  V  F  C  Y  G  H  R  P  F  T  K  Y  P  E
gaaatccctgattacttcaagcaagctttccctgaaggtttaagttgggaaagatctttg
 E  I  P  D  Y  F  K  Q  A  F  P  E  G  L  S  W  E  R  S  L
gaatttgaagacggtggttctgcttcagtttccgcacatataagtttgcgtggtaacaca
 E  F  E  D  G  G  S  A  S  V  S  A  H  M  S  L  R  G  N  T
ttctaccacaagtctaagtttaccggtgttaatttcccagcagatggtcctataatgcaa
 F  Y  H  K  S  K  F  T  G  V  N  F  P  A  D  G  P  M  M  Q
aaccaaagtgtcgactgggaaccatctactgaaaagattacagcttctgatggtgtcttg
 N  Q  S  V  D  W  E  P  S  T  E  K  I  T  A  S  D  G  V  L
aagggtgacgtaactatgtacttgaaattagaaggtggtggtaaccataagtgtcaattc
 K  G  D  V  T  M  Y  L  K  L  E  G  G  G  N  H  K  C  Q  F
aaaactacatacaaggcagccaaaaagatcttgaagatgccaggttcacattacatttcc
 K  T  T  Y  K  A  A  K  K  I  L  K  M  P  G  S  H  Y  I  S
cacagattggttagaaaaacagaaggtaatatcactgaattggttgaagatgctgtcgct
 H  R  L  V  R  K  T  E  G  N  I  T  E  L  V  E  D  A  V  A
cactactctatgttgccttccactagtcatcaccaccatcatcaccatcaccatcacatc
 H  Y  S  M  L  P  S  T  S  H  H  H  H  H  H  H  H  H  H  I
gattgggactactctgaaaaggttaaggaacatttctacaatccaaagaacgccggtgct
 D  W  D  Y  S  E  K  V  K  E  H  F  Y  N  P  K  N  A  G  A
gtagaaggtgcaaacgccattggtgacgttggttcattatcctgtggtgacgctttgaga
 V  E  G  A  N  A  I  G  D  V  G  S  L  S  C  G  D  A  L  R
ttaacattgaaagttgaccctgaaaccgatgtcatcttggacgcaggttttcaaactttc
 L  T  L  K  V  D  P  E  T  D  V  I  L  D  A  G  F  Q  T  F
ggttgcggttctgctattgcatcttcatccgctttgactgaaatggttaagggtttgaca
 G  C  G  S  A  I  A  S  S  S  A  L  T  E  M  V  K  G  L  T
ttggatgaagcattgaaaatctcaaaccaagatatcgctgactatttggatggtttgcca
 L  D  E  A  L  K  I  S  N  Q  D  I  A  D  Y  L  D  G  L  P
cctgaaaagatgcattgttccgtcatgggtagagaagccttacaagctgcagtagctaac
 P  E  K  M  H  C  S  V  M  G  R  E  A  L  Q  A  A  V  A  N
tacagaggtgaaaccattgaagatgaccacgaagaaggtgcattgatatgtaaatgcttt
 Y  R  G  E  T  I  E  D  D  H  E  E  G  A  L  M  C  K  C  F
gccgttgatgaagttatggtcagagataccataagagcaaataagttaagtactgtagaa
 A  V  D  E  V  M  V  R  D  T  M  R  A  N  K  L  S  T  V  E
gatgttactaactacacaaaagctggtggtggttgttctgcttgccatgaagcaatagaa
 D  V  T  N  Y  T  K  A  G  G  G  C  S  A  C  H  E  A  M  E
agagttttgacagaagaattggccgctagaggtgaagtattcgttgcagccccaattaaa
 R  V  L  T  E  E  L  A  A  R  G  E  V  F  V  A  A  P  I  K
gccaaaaagaaagtcaaggtattggctccagaacctgccccagctcctgttgcagaagcc
 A  K  K  K  V  K  V  L  A  P  E  P  A  P  A  P  V  A  E  A
ccagctgcagcccctaagttgtcaaatttgcaaagaattagaagaatcgaaacagtcttg
 P  A  A  A  P  K  L  S  N  L  Q  R  I  R  R  I  E  T  V  L
gctgcaataagacctaccttgcaaagagacaaaggtgacgtcgaattaattgatgtagac
 A  A  M  R  P  T  L  Q  R  D  K  G  D  V  E  L  I  D  V  D
ggtaaaaatgtttacgtcaaattgaccggtgcttgtactggttgccaaatggcatccatg
 G  K  N  V  Y  V  K  L  T  G  A  C  T  G  C  Q  M  A  S  M
acattaggtggtatacaacaaagattgatcgaagaattgggtgagttcgtcaaagttatc
 T  L  G  G  M  Q  Q  R  L  I  E  E  L  G  E  F  V  K  V  I
ccagtctccgctgccgcacacgcccaaatggaagtctgaaagctt
 P  V  S  A  A  A  H  A  Q  M  E  V  *
```

c. CYTOPLASMIC VERSIONS

GAL10p::*flag-nifM* in pESC-Leu

ggccgcatggattacaaggatgacgatgacaagattgcatccgaaagattagccgatggt
M **D Y K D D D K I** A S E R L A D G
gacagtagatattacttgttaaaagttgcccatgaacaatttggttgcgctcctggtgaa
D S R Y Y L L K V A H E Q F G C A P G E
ttatccgaagaacaattgcaacaagctgatagaattataggtagacaaagacacatagaa
L S E E Q L Q Q A D R I M G R Q R H M E
gatgcagttttaagatctccagacgccataggtgttgtcatcccaccttcacaattggaa
D A V L R S P D A M G V V I P P S Q L E
gaagcatgggcccatattgcatctagatacgaatcacctgaagctttgcaacaagcatta
E A W A H I A S R Y E S P E A L Q Q A L
gatgctcaagcattggacgctgctggtatgagagccatgttggctagagaattaagagtc
D A Q A L D A A G M R A M L A R E L R V
gaagctgtattagattgtgtctgcgcaggtttgccagaaatatccgatactgacgttagt
E A V L D C V C A G L P E M S D T D V S
ttgtactacttcaaccatgctgaacaattcaaagtaccagcccaacataaggctagacac
L Y Y F N H A E Q F K V P A Q H K A R H
atttggttaccataaatgaagactttcctgaaaacactagagaagccgctagaactaga
I L V T M N E D F P E N T R E A A R T R
atcgaaacaatcttgaagagattgagaggtaaaccagaaagattcgcagaacaagccatg
I E T I L K R L R G K P E R F A E Q A M
aaacactctgaatgtcctacagctatgcaaggtggtttgttaggtgaagtagttccaggt
K H S E C P T A M Q G G L L G E V V P G
accttgtatcctgaattagatgcttgcttgtttcaaatggcacgtggtgaattatctcca
T L Y P E L D A C L F Q M A R G E L S P
gttttggaatcacctatcggtttccatgttttatactgtgaatctgtctcaccagccaga
V L E S P I G F H V L Y C E S V S P A R
caattgacattggaagaaatcttgcctagattgagagatagattgcaattgagacaaaga
Q L T L E E I L P R L R D R L Q L R Q R
aaggcatatcaaagaaaatggttagaatccttattacaacaaaacgccaccttagaaaac
K A Y Q R K W L E S L L Q Q N A T L E N
ttagcacacggttaagagctc
L A H G *

GAL1p::*his*$_{10}$-*nifH* in pESC-Leu

```
gtcgacatgcatcatcaccatcaccatcaccatcaccatatggccatgagacaatgtgct
       M  H  H  H  H  H  H  H  H  H  H  M  A  M  R  Q  C  A
atctacggtaaaggtggtattggtaaaagtacaaccactcaaaacttggttgccgcttta
 I  Y  G  K  G  G  I  G  K  S  T  T  T  Q  N  L  V  A  A  L
gcagaaatgggtaaaaaggtcatgattgttggttgcgatcctaaggccgactcaactaga
 A  E  M  G  K  K  V  M  I  V  G  C  D  P  K  A  D  S  T  R
ttgatattacactccaaagctcaaaatacaatcatggaaatggcagccgaagcaggtacc
 L  M  L  H  S  K  A  Q  N  T  I  M  E  M  A  A  E  A  G  T
gttgaagatttggaattggaagacgtattgaaggccggttatggtggtgtaaaatgtgtc
 V  E  D  L  E  L  E  D  V  L  K  A  G  Y  G  G  V  K  C  V
gaatctggtggtcctgaaccaggtgtcggttgcgcaggtagaggtgttataactgccatc
 E  S  G  G  P  E  P  G  V  G  C  A  G  R  G  V  M  T  A  I
aatttcttggaagaagaaggtgcatacgaagatgacttagatttcgttttctacgatgta
 N  F  L  E  E  E  G  A  Y  E  D  D  L  D  F  V  F  Y  D  V
ttgggtgacgtcgtttgtggtggttttgctatgccaattagagaaaacaaggcacaagaa
 L  G  D  V  V  C  G  G  F  A  M  P  I  R  E  N  K  A  Q  E
atctatatagtttgcagtggtgaaatgatggctatgtacgctgcaaacaacatctctaag
 I  Y  M  V  C  S  G  E  M  M  A  M  Y  A  A  N  N  I  S  K
ggtatagtcaagtacgccaattctggttcagttagattgggtggtttgatctgtaactca
 G  M  V  K  Y  A  N  S  G  S  V  R  L  G  G  L  I  C  N  S
agaaacactgatagagaagacgaattgattatagccttagctaacaaattgggtacacaa
 R  N  T  D  R  E  D  E  L  I  M  A  L  A  N  K  L  G  T  Q
atgatccatttcgtacctagagataatgtagtccaaagagctgaaatcagaagaatgaca
 M  I  H  F  V  P  R  D  N  V  V  Q  R  A  E  I  R  R  M  T
gttatcgaatatgatccaaaagctaagcaagcagacgaatacagagcattggccagaaag
 V  I  E  Y  D  P  K  A  K  Q  A  D  E  Y  R  A  L  A  R  K
gttgtagataataagttgttagttatacctaacccaatcactatggatgaattggaagaa
 V  V  D  N  K  L  L  V  M  P  N  P  I  T  M  D  E  L  E  E
ttgttgatggaattcggtatcatggaagttgaagatgaatctatagtcggtaaaactgct
 L  L  M  E  F  G  I  M  E  V  E  D  E  S  M  V  G  K  T  A
gaagaagtatgactcgag
 E  E  V  *
```

GAL10p::*flag-nifS* in pESC-Ura

gaattcatggattataaagatgacgatgacaagattgtcgacgccgacgtttacttggat
M **D Y K D D D D K I V** D A D V Y L D
aataacgctactacaagagtcgatgacgaaatagtacaagctatgttgccattttcaca
N N A T T R V D D E M V Q A M L P F F T
gaacaattcggtaacccttccagtttgcattccttcggtaaccaagttggtatggccttg
E Q F G N P S S L H S F G N Q V G M A L
aagaaagctagacaatctgtccaaaaattgttaggtgcagaacacgattccgaaatcgtt
K K A R Q S V Q K L L G A E H D S E I V
tttaccagttgtggtactgaatctgactcaaccgccattttgtctgccttaaaagctcaa
F T S C G T E S D S T A I L S A L K A Q
ccagaaagaaagactgtcataaccactgttgtcgaacatcctgcagtattgtctttatgc
P E R K T V M T T V V E H P A V L S L C
gattatttggcctcagaaggttacactgttcataagttaccagtcgataaaaagggtaga
D Y L A S E G Y T V H K L P V D K K G R
ttggacttagaacactatgcttccttgttaacagatgacgtagctgtagttagtgttatg
L D L E H Y A S L L T D D V A V V S V M
tgggcaaataacgaaactggtacattgtttccaattgaagaaatggcaagattagccgat
W A N N E T G T L F P I E E M A R L A D
gacgctggtataatgttccatactgatgcagtacaagccgttggtaaagtccctatagac
D A G M M F H T D A V Q A V G K V P M D
ttgaagaactcgtcaatccacatgttgtccttaagtggtcataaattgcacgctccaaag
L K N S S I H M L S L S G H K L H A P K
ggtgttggtgtcttgtacttaagaagaggtacaagattcagacctttgttaagaggtggt
G V G V L Y L R R G T R F R P L L R G G
catcaagaaagaggtagaagagccggtactgaaaatgctgcatctattataggtttgggt
H Q E R G R R A G T E N A A S I M G L G
gttgccgctgaaagagctttacaattcatggaacatgaaaacactgaagttaagagattg
V A A E R A L Q F M E H E N T E V K R L
cgtgataagttagaagcaggtattttggccgtcgtaccacacgcatttgttactggtgac
R D K L E A G I L A V V P H A F V T G D
ccagacaatagattacctaacacagctaacatcgcattcgaatacatcgaaggtgaagct
P D N R L P N T A N I A F E Y I E G E A
atcttgttgttgttgaacaaagttggtatagcagcctccagtggttctgcttgtacatct
I L L L L N K V G M A A S S G S A C T S
ggttcattggaaccatcacatgttatgagagcaatggatattccttatacagctgcacac
G S L E P S H V M R A M D I P Y T A A H
ggtactgttagattttctttgagtagatacacaaccgaagaagaaattgatagagtcatt
G T V R F S L S R Y T T E E E I D R V I
agagaagtaccacctattgttgctcaattgagaaaattgtctccttactggtcaggtaat
R E V P P I V A Q L R K L S P Y W S G N
ggtcctgttgaagaccctggtaaagcctttgctcctgtctatggttgaagatct
G P V E D P G K A F A P V Y G *

GAL1p::*his*$_{10}$-*nifU* in pESC-Ura

```
ggatccatgcatcaccaccatcatcaccatcaccatcacatcgattgggactactctgaa
      M  H  H  H  H  H  H  H  H  H  H  I  D  W  D  Y  S  E
aaggttaaggaacatttctacaatccaaagaacgccggtgctgtagaaggtgcaaacgcc
 K  V  K  E  H  F  Y  N  P  K  N  A  G  A  V  E  G  A  N  A
attggtgacgttggttcattatcctgtggtgacgctttgagattaacattgaaagttgac
 I  G  D  V  G  S  L  S  C  G  D  A  L  R  L  T  L  K  V  D
cctgaaaccgatgtcatcttggacgcaggttttcaaactttcggttgcggttctgctatt
 P  E  T  D  V  I  L  D  A  G  F  Q  T  F  G  C  G  S  A  I
gcatcttcatccgctttgactgaaatggttaagggtttgacattggatgaagcattgaaa
 A  S  S  S  A  L  T  E  M  V  K  G  L  T  L  D  E  A  L  K
atctcaaaccaagatatcgctgactatttggatggtttgccacctgaaaagatgcattgt
 I  S  N  Q  D  I  A  D  Y  L  D  G  L  P  P  E  K  M  H  C
tccgtcatgggtagagaagccttacaagctgcagtagctaactacagaggtgaaaccatt
 S  V  M  G  R  E  A  L  Q  A  A  V  A  N  Y  R  G  E  T  I
gaagatgaccacgaagaaggtgcattgatatgtaaatgctttgccgttgatgaagttatg
 E  D  D  H  E  E  G  A  L  M  C  K  C  F  A  V  D  E  V  M
gtcagagataccataagagcaaataagttaagtactgtagaagatgttactaactacaca
 V  R  D  T  M  R  A  N  K  L  S  T  V  E  D  V  T  N  Y  T
aaagctggtggtggttgttctgcttgccatgaagcaatagaaagagttttgacagaagaa
 K  A  G  G  G  C  S  A  C  H  E  A  M  E  R  V  L  T  E  E
ttggccgctagaggtgaagtattcgttgcagccccaattaaagccaaaaagaaagtcaag
 L  A  A  R  G  E  V  F  V  A  A  P  I  K  A  K  K  K  V  K
gtattggctccagaacctgccccagctcctgttgcagaagccccagctgcagcccctaag
 V  L  A  P  E  P  A  P  A  P  V  A  E  A  P  A  A  A  P  K
ttgtcaaatttgcaaagaattagaagaatcgaaacagtcttggctgcaataagacctacc
 L  S  N  L  Q  R  I  R  R  I  E  T  V  L  A  A  M  R  P  T
ttgcaaagagacaaaggtgacgtcgaattaattgatgtagacggtaaaaatgtttacgtc
 L  Q  R  D  K  G  D  V  E  L  I  D  V  D  G  K  N  V  Y  V
aaattgaccggtgcttgtactggttgccaaatggcatccatgacattaggtggtatacaa
 K  L  T  G  A  C  T  G  C  Q  M  A  S  M  T  L  G  G  M  Q
caaagattgatcgaagaattgggtgagttcgtcaaagttatcccagtctccgctgccgca
 Q  R  L  I  E  E  L  G  E  F  V  K  V  I  P  V  S  A  A  A
cacgcccaaatggaagtctgaaagctt
 H  A  Q  M  E  V  *
```

Supplementary Figure 4. Synthetic constructs for the expression of codon-optimized *A. vinelandii nifH*, *nifM*, *nifU*, and *nifS* genes in *S. cerevisiae*. Nucleotides and the corresponding amino acid sequences are aligned. Restriction site sequences used to clone these constructs into pESC expression vectors are double underlined; mitochondrial leader sequences (MLS) are shown in italics; the proposed sites for MLS cleavage are underlined (when the exact cleavage site is known, this is indicated by bold underlined characters within the MLS); FLAG or his-tag amino acid sequences are represented in bold. The mKO and yEGFP amino acid sequences are represented in orange and green, respectively. The flexible linker and tetracysteine motif are shown in red in blue, respectively. Nif amino acid sequences are represented in black.

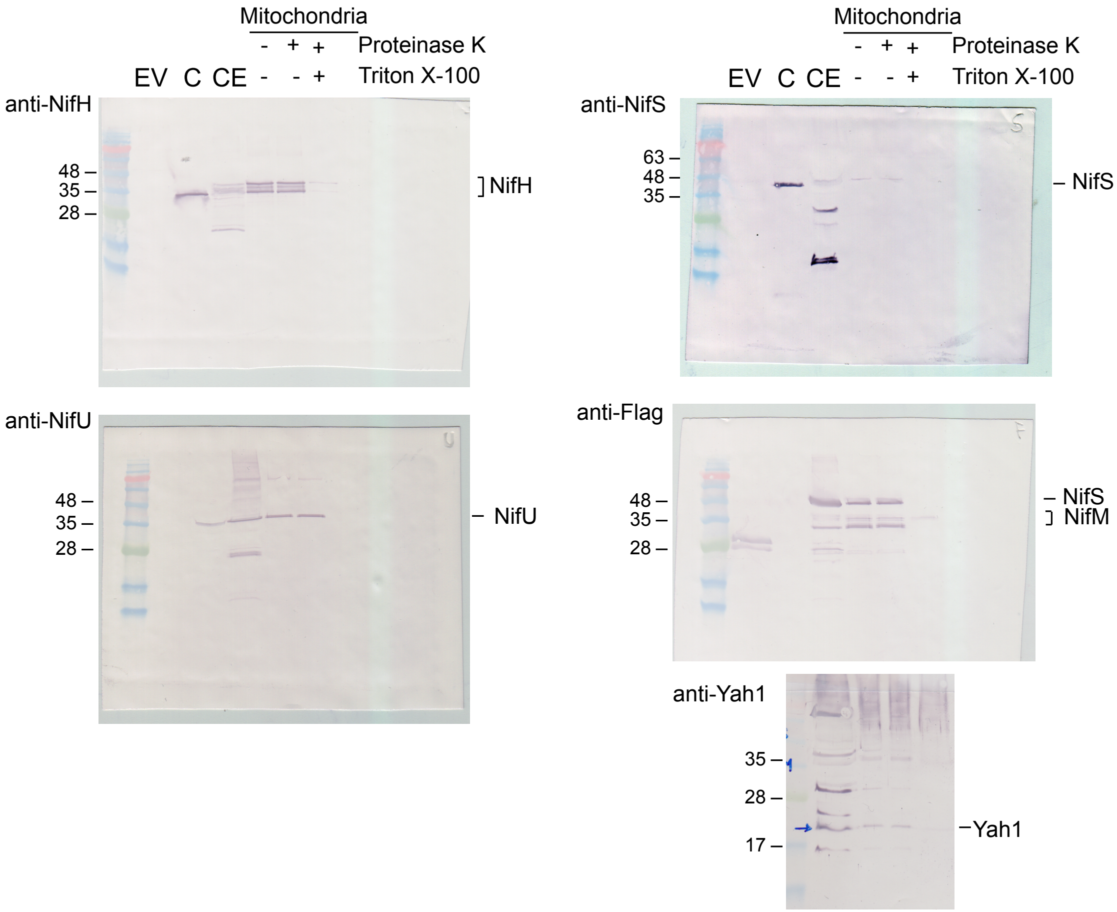

Supplementary Figure 5. Immunoblot analysis of isolated mitochondria. Full blots of those shown in Figure 1b developed with antibodies against NifH, NifU, NifS, Yah1, or FLAG (to detect NifS and NifM at the same time). EV, cell-free extracts from recombinant yeast carrying pESC-His and pESC-Ura plasmids. CE, cell-free extracts from recombinant yeast carrying NifH, NifM, NifU, and NifS cloned into pESC-His and pESC-Ura plasmids (strain GF8). C, Control lanes with purified Nif proteins from *A. vinelandii*. Isolated Mitochondria were treated with Proteinase K either in absence of detergent (for the removal of outer membrane proteins), or in presence of Triton X-100 (to permeabilize the organelle).

Supplementary Table 1. List of strains used in this work.

Strain	Genotype and origin	Source
A. vinelandii		
DJ	Wild type	[5]
DJ33	*ΔnifDK*	[6]
E. coli		
DH5α	F^-ϕ80Δ*lacZM15* Δ(*lacZYA-argF*)*U169 deoP recA1 endA1 hsdR17* (r_K^- m_K^-)	[7]
BL21	F^- (*ompT* $r_B^- m_B^-$)	Novagene
BL21 pRHB609	F^- (*ompT* $r_B^- m_B^-$), T7l$_{ac}$::*his-tev-nifUnifS*	This work
S. cerevisiae		
W303-1a	*MAT*a {*leu2-3, 112 trp1-1 can1-100 ura3-1 ade2-1 his3-11,15*}	ATCC
GF1	W303-1a GAL10p::*mlsSOD2-flag-nifM* in pESC-His	This work
GF2	W303-1a GAL1p::*mlsSOD2-his$_7$-nifH* and GAL10p::*mlsSOD2-flag-nifM* in pESC-His	This work
GF3	W303-1a GAL1p::*mlsSOD2-gfp-nifH-cys$_4$* in pESC-Leu	This work
GF4	W303-1a GAL10p::*mlsSOD2-cys$_4$-nifM-mKO* in pESC-His	This work
GF5	W303-1a GAL1p::*mlsSOD2-gfp-nifH-cys$_4$* in pESC-Leu and GAL10p::*mlsSOD2-cys$_4$-nifM-mKO* in pESC-His	This work
GF6	W303-1a GAL1p::*mlsSu9-his$_{10}$-nifU* and GAL10p::*mlsSu9-flag-nifS* in pESC-Ura	This work
GF7	W303-1a GAL1p::*mlsSOD2-gfp-nifS-cys$_4$* and GAL10p::*mlsSOD2-cys$_4$-nifU-mKO* in pESC-Ura	This work
GF8	W303-1a GAL1p::*mlsSOD2-his$_7$-nifH* and GAL10p::*mlsSOD2-flag-nifM* in pESC-His, and GAL1p::*mlsSu9-his$_{10}$-nifU* and GAL10p::*mlsSu9-flag-nifS* in pESC-Ura	This work
GF9	W303-1a GAL1p::*his$_{10}$-nifH* and GAL10p:: *flag-nifM* in pESC-Leu	This work
GF11	W303-1a GAL1p::*mlsSOD2-his$_7$-nifH* and GAL10p::*mlsSOD2-flag-nifM* in pESC-His, and GAL1p::*mlsSu9-nifU* and GAL10p::*mlsSu9-nifS* in pESC-Ura	This work
GF12	W303-1a GAL1p::*mlsSOD2-his$_{10}$-nifH* in pESC-Leu	This work
GF13	W303-1a GAL1p::*his$_{10}$-nifH* and GAL10p:: *flag-nifM* in pESC-Leu, and GAL1p::*his$_{10}$-nifU* and GAL10p::*flag-nifS* in pESC-Ura	This work

Supplementary Methods

Strain growth and culture media. *Saccharomyces cerevisiae* W303-1a (*MAT*a {*leu2-3,112 trp1-1 can1-100 ura3-1 ade2-1 his3-11,15*}) and derivative strains constructed herein were grown at 30ºC and 200 rpm in YPD medium (1% yeast extract, 2% bactopeptone, 2% glucose) or in minimal SD medium (7.7% yeast-nitrogen base and 2% glucose) supplemented with auxotrophic requirements[1]. *Escherichia coli* DH5α was used for storage and amplification of yeast expression vectors. *E. coli* strains were grown at 37ºC in Luria-Bertani medium supplemented with 150 μg/ml ampicillin. *Azotobacter vinelandii* DJ and DJ33 strains were cultivated in Burk's modified medium supplemented with 29 mM ammonium acetate at 30ºC with shaking (200 rpm). Nitrogenase derepression was carried out as described[2].

DNA constructs and generation of *S. cerevisiae* strains. *A. vinelandii nifH, nifM, nifU and nifS* genes were synthesized with codon optimization for *S. cerevisiae* (GenScript, USA). The N-terminal end of *nifH* carries DNA sequences encoding a duplicated superoxide dismutase mitochondrial leader signal from *S. cerevisiae* (*mlsSOD2)* and a seven-histidine tag. The *nifM* gene carries an N-terminal *mlsSOD2* followed by sequences encoding a Flag tag. The N-terminal end of *nifU* carries DNA sequences encoding the mitochondrial leader signal from *Neurospora crassa* subunit 9 of mitochondrial F0-ATPase (*mlsSu9)* and a ten-histidine tag. The *nifS* gene carries an N-terminal *mlsSu9* followed by sequences encoding a FLAG tag.

To determine NifM, NifH, NifU and NifS subcellular localization, DNA constructs were synthesized which included a tetracysteine motif (*cys_4*), and sequences encoding yEGFP fused to NifH and NifS, or mKO fused to NifM and NifU. Synthetic

constructs were cloned into the dual multicloning sites of pESC-His vector (*nifH-nifM*) or pESC-Ura vector (*nifU-nifS*) (Agilent Technologies) by standard cloning techniques. DNA construct details are shown in Supplementary Fig. 4. *S. cerevisiae* W303-1a transformation was carried out according to[3]. Generated strains are listed in Supplementary Table 1.

Plasmid pRHB609 was constructed to overproduce *A. vinelandii* NifU and NifS in *E. coli*. The *A. vinelandii nifUS* genomic region (2149 bp) was amplified by PCR with primers 5'-ATGCCATATGTGGGATTATTCGGAAAAGTC-3'and 5'-ATGCGGATCCTCAGCCGTAGACCGGAGCGA-3'and cloned into the *Nde*I and *BamH*I sites of expression vector pET16b-TEV.

Expression and purification of NifU and NifS in *E. coli*. Strain BL21 (pRHB609) cells were grown at 37ºC and 200 rpm in 4-1 batches of LB supplemented with 150 μg/ml ampicillin to an OD_{600} of 0.7. EcNifU and EcNifS expression was induced by adding 1 mM of IPTG and $Fe(NH_4)_2(SO_4)_2$ (0.1 mg Fe per liter) during 14 hours at 18ºC and 150 rpm. Cells were collected by centrifugation at 4ºC and 5,000 rpm for 10 min. Recovered cell paste was frozen and stored into liquid N_2 until used. In a typical purification, *E. coli* BL21 (pRHB609) cell paste was resuspended in anaerobic buffer (50 mM Tris-HCl pH 7.4, 200 mM NaCl, 5 mM β-mercaptoethanol) supplemented with 0.2 mM PMSF, 1 μg/ml leupeptine, and 5 μg/ml DNaseI. Cells were lysed under anaerobic conditions in an Emulsiflex-C5 homogenizer at 15,000 lb/in^2, centrifuged at 50,000 x *g* for 1 h at 4ºC, and filtered with a 0.2 μm pore-size MES filter to eliminate cell debris. Cell-free extracts were supplemented with 0.5 mM of L-cysteine, 2 mM of $Fe(NH_4)_2(SO_4)_2$, and 2 mM of β-mercaptoethanol, and incubated for 1 h at room temperature. EcNifU was purified as

described above for yNifU but using instead Co^{2+} affinity chromatography resins. EcNifS was purified as previously described[4].

References

1 Sherman, F., Fink, G. R. & Hicks, J. B. *Methods in yeast genetics*. (Cold Spring Harbor Laboratory Press, 1986).

2 Shah, V. K., Davis, L. C. & Brill, W. J. Nitrogenase. I. Repression and derepression of the iron-molybdenum and iron proteins of nitrogenase in *Azotobacter vinelandii*. *Biochim. Biophys. Acta* **256**, 498-511 (1972).

3 Chen, W. M., Chang, A. C., Shie, B. J., Chang, Y. H. & Chang, N. C. Molecular cloning and characterization of a mouse alpha 2C2 adrenoceptor subtype gene. *Biochim. Biophys. Acta* **1171**, 219-223 (1992).

4 Zheng, L., White, R. H., Cash, V. L., Jack, R. F. & Dean, D. R. Cysteine desulfurase activity indicates a role for NIFS in metallocluster biosynthesis. *Proc. Natl. Acad. Sci. U. S. A.* **90**, 2754-2758 (1993).

5 Jacobson, M. R. *et al.* Biochemical and genetic analysis of the *nifUSVWZM* cluster from *Azotobacter vinelandii*. *Mol. Gen. Genet.* **219**, 49-57 (1989).

6 Robinson, A. C., Burgess, B. K. & Dean, D. R. Activity, reconstitution, and accumulation of nitrogenase components in *Azotobacter vinelandii* mutant strains containing defined deletions within the nitrogenase structural gene cluster. *J. Bacteriol.* **166**, 180-186 (1986).

7 Sambrook, J. & Russell, D. W. *Molecular cloning. A laboratory manual.* Third edn, (Cold Spring Harbor Laboratory Press, 2001).
